# Supplementary material for: Are there inequalities in the attendance and effectiveness of behavioural weight management interventions for adults in the UK? Protocol for an individual participant data (IPD) meta-analysis
Source: BMJ Open. 2023 Mar 23;13(3):e067607. doi: 10.1136/bmjopen-2022-067607 (PMC10039995; doi:10.1136/bmjopen-2022-067607)
Supplement: Supplementary data [file bmjopen-2022-067607supp001.pdf]

Table 1 Summary of eligible studies for inclusion in IPD meta-analysis

Study name (first author)	Year published	Intervention group description	Control group description	Participant eligibility criteria
**WRAP (Ahern)**	2017	Participants were given vouchers to attend WW (previously Weight Watchers) meetings once a week and access WW digital tools for the duration of their intervention (12- or 52-weeks) for free.	A printed booklet of self-help weight-management strategies (British Heart Foundation).	Inclusion: Aged $\geq$18 years and BMI $\geq$28 kg/m^2.<br><br>Exclusion: Planned or current pregnancy, previous or planned bariatric surgery, current participation in a weight-loss programme, having an eating disorder, non-English speaking.
**BeWEL (Anderson)**	2014	12-month intervention delivered by trained lifestyle counsellors in 3 x 1-hour one-to-one visits during the first 3 months, followed by 9 monthly 15-minute telephone conversations, leading to a total contact time of 5.25 hours.	A printed booklet of self-help weight-management strategies (British Heart Foundation).	Inclusion: Aged 50 to 74 years, had undergone polypectomy for adenoma, and BMI >25 kg/m^2.<br><br>Exclusion: Pregnancy, insulin dependent diabetes mellitus, and any cancer diagnosis.
**BWeL (Aveyard)**	2016	General Practitioners offered participants referral to a commercial weight management programme of 12 1-hour sessions (Slimming World, Rosemary Conley) and gave vouchers to allow them to attend for free.	General Practitioners advised participants to lose weight	Inclusion: Aged $\geq$ 18 years, BMI $\geq$25 kg/m^2 (if Asian ethnicity) or BMI $\geq$30 kg/m^2 (if other ethnicities), have a raised body fat percentage.<br><br>Exclusion: Planned or current pregnancy, previous bariatric surgery, completed or participating in a weight management programme within previous 3 months, non-English speaking.
**Ten Top Tips *[10TT]* (Beeken)**	2017	10TT was a self-guided leaflet-based intervention that used habit-formation theory to aid weight loss. A logbook was provided for participants to self-monitor target behaviours.	Usual care, dependent on the participant's General Practitioner. May include dietary advice or referral to a commercial programme.	Inclusion: Aged $\geq$ 18 years, BMI $\geq$ 30 kg/m^2.<br><br>Exclusion: Unable to provide informed consent due to mental incapacity or active psychotic illness, pregnant, or terminally ill.

**PODOSA (Bhopal)**	2014	15 visits from a dietitian over 3 years, where the dietitian would advise participants on achieving weight loss through culturally adapted and translated resources.	4 visits from a dietitian over 3 years where standard advice on healthy eating, diabetes prevention and physical activity was given.	Inclusion: Aged $\geq$ 35 years, self-identified men and women of Indian or Pakistani origin with waists measuring $\geq$ 90cm (men) or $\geq$ 80cm (women), impaired glucose tolerance or impaired fasting glucose tolerance, the family cook was cooperative.<br><br>Exclusion: Receiving long-term oral corticosteroids or weight loss medication, having long-term health disorders making adherence improbable, pregnant, and unlikely to remain in the UK for 3 years.
**Waste the Waist (Greaves)**	2015	4 x 2-hour group-based sessions in the first month to support behaviour change for weight loss, then 5 x 90-minute group sessions over the next 8 months to support maintenance of behaviour change, totalling 13.5 hours of contact time.	Participants were provided written information on the effects of diet and physical activity on cardiovascular risk.	Inclusion: Aged 40-74 years, BMI $\geq$ 28 kg/m^2, and having a high cardiovascular risk defined using either the Framingham or QRISK2 algorithm.<br><br>Exclusion: Existing heart disease, type 2 diabetes mellitus, BMI > 40 kg/m^2,
**Football Fans in Training (Hunt)**	2014	12 weekly sessions of 90-minutes in length, delivered at 13 Scottish professional football club stadiums. Each 90 min session combined advice on healthy diet with physical activity. The balance of classroom and physical activity sessions changed during the 12 weeks; later weeks focused on physical activity as men became fitter, and the shorter classroom sessions focused on revision. The 12-week active phase was followed by a weight maintenance phase with six post-	12 month waiting list to receive the FFIT intervention.	Inclusion: Men, aged 35-65 years, BMI $\geq$ 28 kg/m^2, completed physical activity readiness questionnaire, not taken part in FFIT previously.<br><br>Exclusion: Blood pressure that contraindicated vigorous exercise (systolic ≥160 mm Hg or diastolic ≥100 mm Hg) were excluded from the more intense physical activity programme sessions.

		programme email prompts during 9 months and a group reunion at the club 6 months after the end of the sessions.		
**(Jebb)**	2011	Participants were given vouchers to attend WW (previously Weight Watchers) meetings once a week for 12 months and access digital tools for free.	Advice from their GP and other standard care in line with national treatment guidelines.	Inclusion: Aged ≥ 18 years, BMI 27-35 kg/m^2, and at least one risk factor for obesity related disease (such central adiposity, type 2 diabetes mellitus without inulin treatment, family history of diabetes).<br><br>Exclusion: Achieved weight loss of ≥ 5kg in previous 3 months, history of clinically diagnosed eating disorder, orthopaedic limitations preventing regular physical activity, untreated thyroid disease or more than one change in thyroid treatment in the previous 6 months; receiving treatment with effects on weight or appetite; gastrointestinal disorders; previous surgical procedure for weight loss; major surgery in the previous 3 months; pregnancy or lactation; insulin-treated diabetes; diabetes diagnosis in the previous 6 months; glycated haemoglobin (HbA1c) of at least 75 mmol/mol (9·0%); heart problems in the previous 3 months; uncontrolled hypertension; new prescription drug for a chronic disorder in the previous 3 months or change in dose in the previous 1 month; history or presence of cancer, with the exception of completely resected basal or squamous cell carcinoma if treatment completed 6 months before enrolment or if treatment was stable; or participation in another clinical trial in the previous 30 days.
**Lighten Up (Jolly)**	2011	In addition to 12 vouchers for free entrance to a local leisure centre, participants were randomised to one of 7 intervention groups (all 12 weeks in	12 vouchers for free entrance to a local leisure centre.	Inclusion: Registered with general practices in the South Birmingham Primary Care Trust, aged ≥ 18 years, had a raised BMI recorded in primary care notes within previous 15 months (White Europeans and all ethnic groups apart from South Asians

		length): Weight Watchers, Slimming World, Rosemary Conley, Size Down, GP-led one-to-one counselling, pharmacy-led one-to-one counselling, or a choice of any of the six intervention programmes.		with no comorbidities BMI 30 $\geq$ kg/m^2, White Europeans and all ethnic groups apart from South Asians with comorbidities BMI 28 $\geq$ kg/m^2, South Asians with no comorbidities 25 $\geq$ kg/m^2, South Asians with comorbidities 23 $\geq$ kg/m^2).<br><br>Exclusion: Unable to understand English or were pregnant.
**POWeR+ (Little)**	2016	Two intervention groups, both consisting of a 24-session web-based weight management programme lasting 6 months.<br><br>POWeR+F provided three scheduled (and four optional) face-to-face nurse support sessions.<br><br>POWeR+R included three phone or email contacts and two optional phone or email contacts.	Brief advice web-pages for a healthier diet.	Inclusion: Aged $\geq$ 18 years and BMI $\geq$ 30 kg/m2 or more (or ≥ 28 kg/m2 with hypertension, hypercholesterolaemia, or diabetes).<br><br>Exclusion: Severe mental health problems, too ill to participate in the study or unable to change diet due to health, pregnant or breastfeeding, perceived inability to walk 100m, another member of household participating, no regular access to the internet.
**(Moore)**	2003	Intervention was targeted at general practitioners and practice nurses, and the unit of randomisation was primary care practice. The intervention consisted of 3x90-minute sessions that trained the practitioners on a model approach to obesity treatment.	Control practices were asked to provide usual care to patients.	Inclusion: Aged 16-64 years and BMI $\geq$ 30 kg/m^2.
**CAMWEL (Nanchahal)**	2012	One-to-one programme delivered across 14 visits over 12 months by advisors trained in obesity causes, diet and physical activity, behaviour change strategies,	Usual care.	Inclusion: Aged $\geq$ 18 years and BMI $\geq$25 kg/m^2.

		motivational interviewing and cognitive behavioural therapy techniques.		
**EDIPS (Penn)**	2009	Participants received regular individual advice from a dietitian and physiotherapist trained in motivational interviewing. Participants were also invited to group events, such as 'cook and eat'. Individual sessions were for 30 minutes monthly for the first 3 months and then every 3 months for up to 5 years.	Brief advice and usual care from GP.	Inclusion: Aged >40 years, BMI > 25kg/m^2, and impaired glucose tolerance of ≥ 7.8 mmol/l and < 11.1 mmol/l.<br><br>Exclusion: Previous diagnosis of diabetes mellitus, chronic illness that makes participation in moderate physical activity impossible, or on a special diet for medical reasons.
**NULevel (Sniehotta)**<br><br>***Weight loss maintenance***	2019	Intervention was delivered via a combination of a single face-to face meeting and regular text messaged (at least 1 every 2 days). The text messages consisted of content that was triggered by participants daily self-weighing and questionnaire completion.	Brief lifestyle advice received by text message on 4 occasions, 3 months apart.	Inclusion: Aged $\geq$ 18 years, BMI $\geq$ 30kg/m^2 ($\geq$ 28kg/m^2 if of South Asian descent) in the 24 months preceding trial, and had lost $\geq$5% of body weight in the preceding 12 months.<br><br>Exclusion: Lost weight through illness or surgical procedures, pregnant or planning to become pregnant during study period, breastfeeding, unable to understand English, diagnosis of an eating disorder or condition that limits physical activity, or plans to leave geographic area during study period.
**DROPLET (Astbury)**	2018	The intervention was a Total Diet Replacement programme, which consisted of weekly behavioural support for 12 weeks and monthly support for 3 months with formula food products providing 810 kcal per day for the first 8 weeks, followed by gradual reintroduction of food.	Behavioural support for weight loss from a practice nurse and a diet programme with modest energy restriction.	Inclusion: Aged $\geq$ 18 years, BMI $\geq$ 30kg/m^2, and participants' GP determines weight loss would benefit health.<br><br>Exclusion: Scheduled or previously received bariatric surgery, currently participating in a weight management programme, and contraindications to total diet replacement.
**LIMIT (Daley)**	2019	Over the course of a 12-week weight maintenance programme, participants carried out daily self-weighting, received 3	Brief advice leaflet.	Inclusion: Aged $\geq$ 18 years, had lost $\geq$ 5% of their weight by the end of their weight loss programme, owned a mobile or landline

Weight loss maintenance		brief support phone calls delivered by non-specialist call centre staff, and text messages sent every other day for the first 4 weeks and twice weekly for the remaining 8 weeks.		phone that could receive text messages, was able to understand English sufficiently to complete study procedures.<br><br>Exclusion: Pregnant or intending to become pregnant during the study period.
**WILMA (Simpson)**<br><br>***Weight loss maintenance***	2015	Two intervention groups, both comprising of a 12-month intervention. Participants in both intervention groups could attend 4 peer group support sessions lasting 1.5 hours for 4 months following the face-to-face sessions.<br><br>Intensive group – participants received 6 one-to-one face-to-face individually tailored motivational interviewing delivered fortnightly for 3 months (each session listing around 60 minutes). In the remaining 9 months of the intervention, participants received monthly motivational interviewing calls lasting around 20 minutes.<br><br>Less intensive group – participants received two face-to-face motivational interviewing sessions two weeks apart and two motivational interviewing phone calls at 6-months and 12-months.	Brief advice leaflet.	Inclusion: Aged 18-70 years, current or previous BMI of $\geq$ 30 kg/m^2, and intentionally lost $\geq$5% of body weight in previous 12 months.<br><br>Exclusion: Previous bariatric surgery (unless reversed), terminal illness, inability to understand study materials in English, living with another study participant, or currently pregnant.
