# Supplementary material for: Are there inequalities in the attendance and effectiveness of behavioural weight management interventions for adults in the UK? Protocol for an individual participant data (IPD) meta-analysis
Source: BMJ Open. 2023 Mar 23;13(3):e067607. doi: 10.1136/bmjopen-2022-067607 (PMC10039995; doi:10.1136/bmjopen-2022-067607)
Supplement: Author's manuscript [file bmjopen-2022-067607.draft_revisions.pdf]

BMJ Open is committed to open peer review. As part of this commitment we make the peer review history of every article we publish publicly available.

When an article is published we post the peer reviewers' comments and the authors' responses online. We also post the versions of the paper that were used during peer review. These are the versions that the peer review comments apply to.

The versions of the paper that follow are the versions that were submitted during the peer review process. They are not the versions of record or the final published versions. They should not be cited or distributed as the published version of this manuscript.

BMJ Open is an open access journal and the full, final, typeset and author-corrected version of record of the manuscript is available on our site with no access controls, subscription charges or pay-per-view fees (http://bmjopen.bmj.com).

If you have any questions on BMJ Open's open peer review process please email info.bmjopen@bmj.com

BMJ Open

Are there inequalities in the attendance and effectiveness of behavioural weight management interventions for adults in the UK? Protocol for an individual participant data (IPD) meta-analysis

Journal:	*BMJ Open*
Manuscript ID	bmjopen-2022-067607
Article Type:	Protocol
Date Submitted by the Author:	19-Aug-2022
Complete List of Authors:	Birch, Jack; MRC Epidemiology Unit<br>Mueller, Julia; University of Cambridge, MRC Epidemiology unit<br>Sharp, Stephen; University of Cambridge, MRC Epidemiology Unit<br>Logue, Jennifer; Lancaster University, Lancaster Medical School<br>Kelly, Michael; University of Cambridge Department of Public Health and Primary Care, Public Health and Primary Care<br>Griffin, Simon; MRC Epidemiology Unit; University of Cambridge Department of Public Health and Primary Care<br>Ahern, Amy; MRC Epidemiology Unit, University of Cambridge
Keywords:	PUBLIC HEALTH, PRIMARY CARE, PREVENTIVE MEDICINE

SCHOLARONE™
Manuscripts

Are there inequalities in the attendance and effectiveness of behavioural weight management interventions for adults in the UK? Protocol for an individual participant data (IPD) meta-analysis

Authors: Jack M Birch[1], Julia Mueller[1], Stephen J Sharp[1], Jennifer Logue[2], Michael P Kelly[3], Simon J Griffin[1,3], Amy L Ahern[1]

Corresponding author

Jack M Birch

MRC Epidemiology Unit, University of Cambridge School of Clinical Medicine, Box 285 Institute of Metabolic Science, Cambridge Biomedical Campus, CB2 0QQ

jack.birch@mrc-epid.cam.ac.uk

Affiliations: [1]MRC Epidemiology Unit, University of Cambridge; [2]Lancaster Medical School, University of Lancaster [3]Department of Public Health and Primary Care, University of Cambridge.

Key words: obesity, inequalities, individual participant data meta-analysis, weight loss

Word count: 3573

For peer review only

Abstract

Introduction: It is important to identify whether behavioural weight management interventions work well across different groups in the population so health inequalities in obesity are not widened. Previous systematic reviews of inequalities in the attendance and effectiveness of behavioural weight management interventions have been limited because few trials report relevant analyses and heterogeneity in the categorisation of inequality characteristics prevents meta-analysis. An individual participant data meta-analysis (IPD-MA) allows us to re-analyse all trials with available data in a uniform way. We aim to conduct an IPD meta-analysis of UK randomised controlled trials to examine whether there are inequalities in the attendance and effectiveness of behavioural weight interventions.

Methods and analysis: In a recently published systematic review, we identified 18 UK-based randomised controlled trials of primary care-relevant behavioural interventions, conducted in adults living with overweight or obesity, and reporting weight outcomes at baseline and 1-year follow up. The corresponding author of each trial will be invited to contribute data to the IPD-MA. The outcomes of interest are weight change between baseline and 1-year, and intervention attendance (number of sessions offered versus number of sessions attended). We will primarily consider whether there is an interaction between intervention group and characteristics where inequalities occur, such as by sex, socioeconomic status, or age. The IPD-MA will be conducted in line with the Preferred Reporting Items for Systematic Review and Meta-analysis of IPD (PRISMA-IPD) guidelines.

Ethics and dissemination: No further ethical approval was required as ethical approval for each individual study was obtained by the original trial investigators from appropriate ethics committees. The completed IPD-MA will be disseminated at conferences, in a peer-reviewed journal, and contribute to the lead author's PhD thesis. Investigators of each individual study included in the final IPD-MA will be invited to collaborate on any publications that arise from the project.

For peer review only - http://bmjopen.bmj.com/site/about/guidelines.xhtml

Strengths and limitations of this study

- To the best of our knowledge, this will be the first individual participant data meta-analysis examining inequalities in the attendance and effectiveness of behavioural weight management interventions.
- Individual participant data meta-analyses allow for data harmonisation, which increases the amount of data that can be used and the statistical power to explore whether inequalities are present in the attendance and outcome of behavioural weight management interventions.
- The findings of this study may identify groups that these interventions are less effective in, informing future public health research and practice.
- Due to complexities in developing data sharing agreements and harmonisation of inequality variables in acquiring data from other countries, only UK-based trials are included in this individual participant data meta-analysis.

For peer review only

1. Introduction

Inequalities in overweight and obesity are widely recognised – those who experience higher levels of socioeconomic deprivation, especially women, are more likely to live with obesity than those who more affluent.[1,2] Similarly, comorbidities of obesity are more common in those experiencing socioeconomic deprivation.[3] It has also been suggested that interventions focusing on individual behaviour change, such as behavioural weight management interventions, may exacerbate health inequalities.[4,5] These intervention-generated inequalities may occur at different stages, including intervention uptake, attendance and effectiveness.

We recently conducted a systematic review to synthesise evidence on how different measures of inequality moderate the uptake, attendance and effectiveness of behavioural weight management interventions in adults.[6] We found that most trials did not consider whether inequalities were generated in the studied intervention; where these analyses were conducted, most found no evidence of inequalities. Where an inequalities gradient was observed, intervention uptake, adherence and attrition generally favoured those considered as 'more advantaged' (such as those who are White, with higher income, or older). Due to substantial differences in the reporting of measures of inequality, together with the low level of reporting of analyses of inequalities, we were unable to perform a quantitative synthesis of the reported results. Hence, it is not possible to fully explore inequalities using aggregated data from published literature alone. This lack of reporting may have occurred because individual trials may not be large enough to detect an interaction between moderators such as socioeconomic status [SES] and the outcome; they are likely just to have been designed to detect an overall effect.

These limitations can be addressed in part by conducting a meta-analysis of individual participant data (IPD), which requires the central collation, aggregation and reanalysis of IPD from relevant trials.[7,8] This allows for data in each study to be analysed and defined in a uniform way, overcoming heterogeneity issues associated with using aggregate data. Meta-analysis of IPD may provide sufficient

For peer review only - http://bmjopen.bmj.com/site/about/guidelines.xhtml

For peer review only

statistical power to consider whether there are inequalities in uptake, attendance and effectiveness of interventions.[7,9]

1.2 Research questions

1. To what extent do the weight outcomes of those who have participated in a behavioural weight management trial differ by individual characteristics that stratify health opportunities and outcomes (as defined using the PROGRESS Plus Framework)?
2. To what extent does the effectiveness of behavioural weight management interventions differ by individual characteristics that stratify health opportunities and outcomes?
3. To what extent does attendance at behavioural weight management interventions differ by individual characteristics that stratify health opportunities and outcomes?

2. Methods and analysis

This IPD meta-analysis responds to limitations identified in our previous systematic review on inequalities in the uptake of, adherence to, and effectiveness of behavioural weight management interventions. The Preferred Reporting Items for Systematic Reviews and Meta-Analyses IPD extension will be followed when reporting this study.[10]

2.1 Trial identification

2.1.1 Search strategy

This study includes UK-based trials of behavioural weight management interventions that we identified through a previous systematic review.[6] We focused on UK-based trials to reduce heterogeneity in measures of the PROGRESS-Plus characteristics and in the context in which the

For peer review only

interventions were delivered. Characteristics such as ethnicity and socioeconomic status are conceptualised differently in different countries which makes synthesising data across these characteristics inappropriate or not possible. For example, socioeconomic status in the UK is often captured using Indices of Multiple Deprivation (IMD), an area-based measure, which is not replicated in other countries. There are also pragmatic reasons for focusing on UK-based trials; the complexity of arranging cross-country data sharing would have made the timelines for this project unviable.

Studies published since the search strategy in the systematic review was conducted, were identified through an updated Medline search and through discussions with the corresponding authors of the included trials. The inclusion and exclusion criteria we used to identify relevant trials for this IPD meta-analysis are:

1. Participants: adults aged 18 years and over with overweight or obesity (body mass index [BMI] > 25 kg/m2 with no upper limit) who were deemed suitable (either by the applicable study team or healthcare practitioner) for weight loss or weight loss maintenance intervention. Participants may have additional risk factors such as hypertension, dyslipidaemia, impaired glucose tolerance or impaired fasting glucose. Studies were excluded if the population was not selected based on a weight-related measure, included participants with BMI<25 kg/m2, were selected based on having a chronic disease where weight loss is part of disease management or being pregnant, or if the intervention was targeted at parents to change behaviour of children. Only studies conducted in the UK were eligible.
2. Interventions: behavioural weight management interventions with the primary aim of supporting weight loss or weight loss maintenance. Studies were included if they were conducted in, or were applicable to, primary care settings. Interventions may have been delivered alone or as part of a wider multicomponent intervention targeting diet and nutrition, physical activity, sedentary behaviour, or a combination of these. Interventions may include, but were not limited to, assessment with feedback, advice, provider training, goal-

For peer review only - http://bmjopen.bmj.com/site/about/guidelines.xhtml

setting, or exercise referral. Studies of pharmacological and surgical interventions were excluded unless the trial included behavioural only and control arms. Interventions were considered feasible for application to primary care if they were conducted in a healthcare setting or were widely available in the community at a national or regional level (such as commercial weight loss programmes, text-message and other digital-based interventions); examples of settings that are not relevant to primary care include interventions delivered in inpatient settings, or in residential care homes.

3. Comparators: wait-list control, usual care, or minimal intervention (such as generic print or electronic materials).
4. Outcomes: studies must report weight change in kg at a time point between the 12- or 18-month follow up.
5. Study designs: randomised or cluster-randomised controlled trials [RCTs].

The trials we identified as being eligible for inclusion are outlined in Supplementary Table 1.[11–28]

2.2 Study outcomes, exposures and covariates

2.2.1 Outcomes

Outcomes are weight (kg) at 12-month follow up and intervention attendance. Where data allows, attendance will be measured as the percentage of offered sessions which were attended.

2.2.2 Exposures

Exposure variables are selected measures of the PROGRESS-Plus criteria where data are likely to be available in UK-based trials of behavioural weight management interventions. Coding of each exposure variable will depend on the variables and coding used in each study providing individual

For peer review only - http://bmjopen.bmj.com/site/about/guidelines.xhtml

participant data. Should the provided variables be suitably homogeneous, then we anticipate that the coding for the IPD meta-analysis will consist of the following:

- Ethnicity (coded using the England and Wales census categories: Asian or Asian British/Black, African, Caribbean or Black British/Mixed or Multiple ethnic groups/Other ethnic group/White/Missing)
- Occupation (Employed/Unemployed/Retired/Other (i.e. student, carer, voluntary work)/Missing)
- Gender (Male/Female/Non-Binary/Not provided or other)
- Religion (coded using the England and Wales census categories: No religion/Christian/Buddhist/Hindu/Jewish/Muslim/Sikh/Other religion/Missing)
- Education (University degree or equivalent, or higher/Post-secondary education/A-levels or equivalent/GCSEs or equivalent/No formal qualifications/Missing)
- SES – Household income (<£40 000/≥£40 000/Missing)
- SES – Indices of Multiple Deprivation [IMD] (1 –most deprived/2/3/4/5 – least deprived/Missing)
- Social capital – Relationship status (Single/Married or co-habiting/Widowed, separated or divorced/Missing)
- Age (Continuous integer values)
- Disability (Has physical- or mental health-related disability/No disability/Missing)
- Randomised group (Control/Intervention)

2.2.3 Covariates

- Baseline weight (kg)

For peer review only - http://bmjopen.bmj.com/site/about/guidelines.xhtml

2.3 Risk of bias assessment

We will use Cochrane's risk of bias tool for randomised controlled trials (RoB 2) to assess the risk of bias in all studies meeting our inclusion criteria.[29] The tool facilitates researchers to consider bias across six domains: the randomisation process; allocation concealment; participant and trial personnel blinding; blinding of outcome assessment; incomplete outcome data; and selective reporting. A rating of 'low risk', 'high risk', or 'unclear' will be assigned to each domain by two contributors independently. Where disagreements occur, these will be resolved by discussion to reach consensus or through consultation with a third contributor. We will present the results of the risk of bias assessment in a summary figure outlining a study's overall risk of bias in addition to the risk of bias in each domain.

2.4 Data collection and management

Our approach to collecting and aggregating the individual participant data was informed by the PRISMA-IPD extension and previously published IPD meta-analysis protocols.[7,10,30–32]

2.4.1 Invitation of authors

Trial investigators of all eligible trials were invited by email, using contact details acquired through trial publications, to contribute data and collaborate on this study. The email outlined our research aims and the specific data we were requesting.

2.4.2 Data collection

Standardised data specification forms will be sent to trial authors. Data will be requested in Microsoft Excel format; however, data will be accepted in any format. Once received, a master copy of each trial dataset will be saved in its original format and preserved. Any non-Microsoft Excel format datasets will be converted and then imported into Stata version 16 (StataCorp. 2019. Stata Statistical Software:

For peer review only - http://bmjopen.bmj.com/site/about/guidelines.xhtml

Release 16.). We will also ask for detailed definitions of the measures used in the trial so we can ensure appropriate harmonisation.

2.4.3 Data checking

Once data are received from trial authors, they will be checked for quality and to ensure they pertain to the correct trial. Descriptive statistics (sample size, demographic variables, weight loss or BMI change) will be performed for each individual trial; should discrepancies occur between our analysis and the original trial publication, then the study authors will be contacted for clarification. Should clarification not be received, then the size of the discrepancy will be considered. If it is small and unlikely to bias the results, then the data will be included in the IPD meta-analysis. Large data inaccuracy and excessive missing data (versus what is reported in the trial publication) may lead to a trial being excluded.

2.4.3 Database creation and aggregation

A single database will be created containing data from all the trials. There is likely to be differences between studies in the coding of measures of inequalities. For example, there is variation in measures of SES that are used. Hence, once all data are received and the differences in coding across the measures become apparent, we will discuss amongst the core research team (JMB, MPK, SJG, and ALA) the best approach to achieve consistent reporting across all measures.

Once the data checking has been completed, variables will be recoded to match the coding of the IPD database. The data from each individual trial will then be copied into the IPD database and checked to ensure the integrity of the data has been maintained through the merge. Each individual trial dataset will be given a unique identifier prior to the merge.

For peer review only

For peer review only - http://bmjopen.bmj.com/site/about/guidelines.xhtml

2.4.4 Trials where IPD are not available

Where we are unable to obtain IPD for an eligible trial, we will ask the trial investigator if they are able to conduct the analyses using the same coding of variables as defined in section 2.5.2 and provide us with the outcome statistics. We will offer this as an option to ensure that we are able to include as much relevant data as possible and we will provide the relevant code to facilitate this. If the outcome statistics are provided from any trials, we will meta-analyse this together with the trials for which we obtained individual participant data. If we synthesise results from data that we did not receive individual participant data for, we will conduct sensitivity analyses excluding these data. Further sensitivity analyses excluding studies with 'high' risk of bias will also be conducted.

2.5 Statistical analysis

Due to our research questions exploring treatment effect and covariate interactions, we decided that a two-stage IPD meta-analysis would be most appropriate. In the first stage, regression analyses are performed individually in each trial. Then in the second stage combines the outcome estimates from each model using a more standard meta-analysis approach (e.g. random-effects meta-analysis).[33] We are using a two-stage approach because it inherently avoids aggregation bias and controls for trial-level confounding, to which one-stage IPD meta-analyses are more susceptible.[33] An additional benefit of performing a two-stage IPD meta-analysis is that trials for which we are unable to acquire individual-level data may still be included in the synthesis provided the relevant outcome statistics can be obtained. Data analysis will be conducted using Stata v16 (StataCorp. 2019. Stata Statistical Software: Release 16. College Station, TX: StataCorp LLC.). The IPD meta-analysis will be performed using the ipdmetan package available in Stata v16. When meta-analysing outcome statistics from trials we received IPD for and trials where these statistics were provided to us, we will use other meta-analysis commands within STATA version 16 to complete these analyses.

For peer review only

2.5.1 Baseline characteristics

We will describe the baseline characteristics for randomised group and each PROGRESS-Plus characteristic. This will be completed for each trial and as an overall aggregate of all participants included in the meta-analysis. We will compare these characteristics descriptively with data on the prevalence of obesity in the population, such as the Health Survey for England and other studies that have considered who routinely accesses behavioural weight management interventions.[34–36]

2.5.2 Analysis of study outcomes

We will conduct six sets of analyses (if there are sufficient data for each outcome), two for each of our research questions, as we will synthesise data on weight loss interventions separately to data for weight loss maintenance interventions. As all outcomes of interest are continuous, we will use multivariable linear regression models, and include the relevant parameter estimates and standard errors from the models in random-effects meta-analyses. Heterogeneity will be assessed using tau-squared, which summarises between-studies variance, and a 95% prediction interval which indicates the range in which 95% of the true effects lie. Inconsistency will be assessed using I-squared, which indicates the proportion of total variability in the observed effects that is due to heterogeneity.

The subgroups used for each exposure variable are listed below (reference subgroup in bold). If free-text responses are available for any 'other' subgroup for each exposure, we will recode to the most appropriate subgroup in that exposure. If this is not possible, we will recode 'other' to missing. 'Prefer not to say' responses will also be recoded to missing. We anticipate that certain subgroups of some variables will likely have few, if any, data. These will be recoded to missing and excluded from the analyses.

- Ethnicity (**White**/Ethnic minorities (excluding White minorities))
- Occupation (Unemployed/**Employed**/Unable to work/Retired/Student)
- Gender (**Female**/Male/Other or Non-binary)
- Religion

For peer review only - http://bmjopen.bmj.com/site/about/guidelines.xhtml

- Education (No formal qualifications/**GCSEs, O-Levels, or equivalent**/A-Levels or equivalent/Some additional training/University degree)
- Index of Multiple Deprivation (**1 – Most deprived**/2/3/4/5 – Least deprived)
- Household income (**<£40,000**/≥£40,000)
- Relationship status (Single/Married or co-habiting/Widowed, separated, or divorced)
- Age (continuous)
- Randomised group (**Control**/Intervention)

We will present summary statistics for weight and attendance outcomes separately for each trial and as combined values across all trials.

2.5.2.1 Research question 1: To what extent do the weight outcomes of those who have participated in a behavioural weight management trial differ by individual characteristics that stratify health opportunities and outcomes?

In these analyses, each trial will be analysed as a cohort study. Using multivariable regression, we will estimate the association between the 'exposure' variable (i.e. each PROGRESS-Plus characteristic we have sufficient data for), and weight at 12-month follow up. Each model will be adjusted for age and gender (with the exception of the models where age and gender are considered as the exposure variables), baseline weight, and assigned intervention. Associations will be estimated for each exposure subgroup within a trial, and these associations will then be meta-analysed across trials.

2.5.2.2 Research question 2: To what extent does the effectiveness of behavioural weight management interventions differ by individual characteristics that stratify health opportunities and outcomes?

These analyses will focus on intervention effects by subgroup. Multivariable linear regression models will be used to test the null hypothesis that there is no interaction between each PROGRESS-Plus characteristic and intervention group on weight at 12 months. Each model will be adjusted for age and gender (with the exception of the models where age and gender are considered as the exposure

For peer review only - http://bmjopen.bmj.com/site/about/guidelines.xhtml

variables) and baseline weight. The intervention effects will be estimated by subgroup within each trial. These subgroup-specific effects will then be meta-analysed across trials.

2.5.2.3 Research question 3: To what extent does attendance of behavioural weight management interventions differ by individual characteristics that stratify health opportunities and outcomes?

For the third research question, each trial will be analysed as a cohort study. Attendance will be considered as a percentage – the number of sessions attended divided by the maximum possible number of sessions a participant could attend – and treated as a continuous variable. Multivariable regression models will be used to estimate the association between each PROGRESS-Plus characteristic and attendance. Each model will be adjusted for age and gender (with the exception of the models where age and gender are considered as the exposure variables), and baseline weight, and assigned intervention. Associations will be estimated for each exposure subgroup within a trial, and these associations will then be meta-analysed across trials.

2.5.6 Missing data

A complete-case analysis will be performed, i.e. participants who have missing data for either the outcome, exposure or covariates will be excluded.

2.5.7 Sensitivity analysis

As highlighted in 2.4.4, if we synthesise results from data that we did not receive individual participant data for, we will conduct sensitivity analyses excluding these data. Sensitivity analyses excluding studies with 'high' risk of bias will also be conducted to consider whether these studies have an impact on the results. Should sufficient data be obtained, we will also conduct further analyses to consider whether intervention characteristics affect inequalities in attendance and effectiveness. These

For peer review only

analyses may include comparisons by intervention length, digital versus non-digital, and group based versus individually based.

2.6 Patient and public involvement

As part of the protocol development for the preceding systematic review, we received comments on a lay summary from a patient and public involvement (PPI) representative on the project aims and our definition of the PROGRESS-Plus characteristics.[37] These aims and definitions have been brought forward into this IPD meta-analysis project. We will seek further PPI input on our harmonisation of the subgroups of the exposure variables to ensure our categorisations are appropriate, and a PPI representative will contribute to the interpretation of data and will co-author the final manuscript.

3. Discussion

There is some evidence from our previously conducted systematic review that those we may consider to be 'more advantaged' (such as having more years of education, higher income, being White, and being older) may be the most likely to maintain attendance to and have better outcomes from behavioural weight management interventions.[6] However, evidence was mixed and in that review we were unable to quantitatively synthesise data on attendance and weight outcomes due to heterogeneity in study populations (our review focused on all Organisation for Economic Co-operation and Development countries) and measures of the PROGRESS-Plus characteristics (for example, race and ethnicity are captured very differently in the US versus the UK). This heterogeneity will be partly addressed in this IPD meta-analysis by focusing on trials from a single country (the UK) and through the data harmonisation that can be achieved when access to IPD is obtained.

This IPD meta-analysis will have several implications for public health policy, practice and research. The analyses may identify certain sociodemographic groups that have lesser attendance, or attain lesser weight loss. From a research perspective, future work could seek to establish why this may be

For peer review only - http://bmjopen.bmj.com/site/about/guidelines.xhtml

the case; for public health policy, it is important to identify groups where interventions may be generating or exacerbating inequalities so additional support or provision can be offered to prevent this from occurring.

3.1 Strengths and limitations

There are several strengths of conducting an IPD meta-analysis in comparison to a conventional meta-analysis. IPD meta-analyses are particularly useful for considering moderators of intervention outcomes,[7] due to the increased statistical power gained by pooling data (although this is not guaranteed for all moderating variables, as it depends on the available data). Harmonisation of variables across studies means more data can be pooled together, leading to more robust analyses and conclusions. A further strength of IPD meta-analyses is that they go beyond published data, which may be limited in the measures reported. Receiving the original trial data also allows for increased data checking and increased validation of previously published results.[8]

However, there are also limitations of conducting an IPD meta-analysis. Even though the raw trial data will be acquired, analysis is dependent on the measures assessed in each original trial, and may be limited. Data harmonisation that is required to conduct an IPD meta-analysis may lead to some data being excluded from the analyses as it is unlikely to be possible to harmonise all data from different measures of each PROGRESS-Plus characteristic. A further limitation is that we are only looking at UK-based trials of behavioural weight management interventions, which may limit the generalisability of our findings to other countries or healthcare systems.

3.2 Ethics and dissemination

Ethical approval was not required for this study as no primary data are to be collected, and the IPD are to be analysed in accordance to the purpose for which they were originally collected for. Ethical approval for each eligible trial for this IPD meta-analysis was obtained by the original investigators of each trial.

For peer review only - http://bmjopen.bmj.com/site/about/guidelines.xhtml

We anticipate that the completed IPD meta-analysis will be published in a scientific journal; one collaborator from each trial contributing IPD will be invited to be a co-author on the publication. The findings from this IPD meta-analysis study may also be presented at relevant public health and obesity research conferences, and will contribute to the lead investigator's PhD thesis.

Contributors:

JMB conceived and designed the study, developed the analysis plan, and drafted the manuscript. JM contributed to study design and the analysis plan, and reviewed drafts of the manuscript. SJS contributed to the analysis plan and reviewed drafts of the manuscript. JL, MPK and SJG contributed to study design and reviewed drafts of the manuscript. ALA conceived and designed the study, contributed to the analysis plan, and reviewed drafts of the manuscript. All authors approved the final version for publication.

Funding:

JMB, ALA, SJG, and SJS are supported by the Medical Research Council (MRC) (Grant MC_UU_00006/6). The University of Cambridge has received salary support in respect of SJG from the National Health Service in the East of England through the Clinical Academic Reserve. This work is funded by UKRI grant MC_UU_00006/6.

Conflicts of interest:

JM is a trustee for the Association for the Study of Obesity (unpaid role). JL has received research support from Slimming World and consulting fees from Novo Nordisk, and is an employee of Astrazeneca. This work was performed before she became an Astrazeneca employee and Astrazeneca had no role in the work. MPK has undertaken consultancy for Slimming World and led the obesity and weight management guidelines development for NICE from 2005 until 2014. SJG is principal

For peer review only

For peer review only - http://bmjopen.bmj.com/site/about/guidelines.xhtml

investigator on a publicly funded (NIHR) trial in which the intervention is provided by WW (formerly Weight Watchers) at no cost. ALA is principal investigator on two publicly funded (NIHR, MRC) trials where the intervention is provided by WW (formerly Weight Watchers) at no cost.

References

1. Newton S, Braithwaite D, Akinyemiju TF. Socio-economic status over the life course and obesity: Systematic review and meta-analysis. *PloS one*. 2017;12(5):e0177151-e0177151.
2. El-Sayed AM, Scarborough P, Galea S. Unevenly distributed: a systematic review of the health literature about socioeconomic inequalities in adult obesity in the United Kingdom. *BMC public health*. 2012;12(1):18.
3. Khanolkar AR, Patalay P. Socioeconomic inequalities in co-morbidity of overweight, obesity and mental ill-health from adolescence to mid-adulthood in two national birth cohort studies. *The Lancet Regional Health – Europe*. 2021;6. doi:10.1016/j.lanepe.2021.100106
4. White M, Adams J, Heywood P. How and why do interventions that increase health overall widen inequalities within populations? In: Babones SJ, ed. *Social Inequality and Public Health*. Policy Press; 2009. https://books.google.co.uk/books?id=NHRoDwAAQBAJ
5. Adams J, Mytton O, White M, Monsivais P. Why Are Some Population Interventions for Diet and Obesity More Equitable and Effective Than Others? The Role of Individual Agency. *PLOS Medicine*. 2016;13(4):e1001990.
6. Birch JM, Jones RA, Mueller J, et al. A systematic review of inequalities in the uptake of, adherence to, and effectiveness of behavioral weight management interventions in adults. *Obesity Reviews*. 2022;23(6):e13438. doi:10.1111/obr.13438
7. Weitz E, Kleiboer A, van Straten A, Hollon SD, Cuijpers P. Individual patient data meta-analysis of combined treatments versus psychotherapy (with or without pill placebo), pharmacotherapy or pill placebo for adult depression: a protocol. *BMJ Open*. 2017;7(2):e013478.
8. Stewart LA, Tierney JF. To IPD or not to IPD? Advantages and disadvantages of systematic reviews using individual patient data. *Eval Health Prof*. 2002;25(1):76-97.
9. Chow CK, Islam SMS, Farmer A, et al. Text2PreventCVD: protocol for a systematic review and individual participant data meta-analysis of text message-based interventions for the prevention of cardiovascular diseases. *BMJ Open*. 2016;6(10):e012723.
10. Stewart LA, Clarke M, Rovers M, et al. Preferred Reporting Items for a Systematic Review and Meta-analysis of Individual Participant Data: The PRISMA-IPD Statement. *Jama*. 2015;313(16):1657-1665.
11. Ahern AL, Wheeler GM, Aveyard P, et al. Extended and standard duration weight-loss programme referrals for adults in primary care (WRAP): a randomised controlled trial. *Lancet (London, England)*. 2017;389(10085):2214-2225. doi:https://dx.doi.org/10.1016/S0140-6736(17)30647-5

For peer review only

12. Anderson AS, Craigie AM, Caswell S, et al. The impact of a bodyweight and physical activity intervention (BeWEL) initiated through a national colorectal cancer screening programme: randomised controlled trial. *BMJ*. 2014;348:g1823. doi:10.1136/bmj.g1823

13. Aveyard P, Lewis A, Tearne S, et al. Screening and brief intervention for obesity in primary care: a parallel, two-arm, randomised trial. *The Lancet*. 2016;388(10059):2492-2500. doi:10.1016/S0140-6736(16)31893-1

14. Beeken RJ, Leurent B, Vickerstaff V, et al. A brief intervention for weight control based on habit-formation theory delivered through primary care: results from a randomised controlled trial. *International journal of obesity (2005)*. 2017;41(2):246-254. doi:10.1038/ijo.2016.206

15. Bhopal RS, Douglas A, Wallia S, et al. Effect of a lifestyle intervention on weight change in south Asian individuals in the UK at high risk of type 2 diabetes: a family-cluster randomised controlled trial. *Lancet Diabetes Endocrinol*. 2014;2(3):218-227. doi:10.1016/s2213-8587(13)70204-3

16. Greaves C, Gillison F, Stathi A, et al. Waste the waist: a pilot randomised controlled trial of a primary care based intervention to support lifestyle change in people with high cardiovascular risk. *International Journal of Behavioral Nutrition and Physical Activity*. 2015;12(1):1.

17. Hunt K, Wyke S, Gray CM, et al. A gender-sensitised weight loss and healthy living programme for overweight and obese men delivered by Scottish Premier League football clubs (FFIT): a pragmatic randomised controlled trial. *The Lancet*. 2014;383(9924):1211-1221. doi:10.1016/S0140-6736(13)62420-4

18. Jebb SA, Ahern AL, Olson AD, et al. Primary care referral to a commercial provider for weight loss treatment versus standard care: a randomised controlled trial. *Lancet*. 2011;378(9801):1485-1492. doi:10.1016/s0140-6736(11)61344-5

19. Jolly K, Lewis A, Beach J, et al. Comparison of range of commercial or primary care led weight reduction programmes with minimal intervention control for weight loss in obesity: lighten Up randomised controlled trial. *BMJ*. 2011;343:d6500. doi:10.1136/bmj.d6500

20. Little P, Stuart B, Hobbs FR, et al. An internet-based intervention with brief nurse support to manage obesity in primary care (POWeR+): a pragmatic, parallel-group, randomised controlled trial. *Lancet Diabetes Endocrinol*. 2016;4(10):821-828. doi:10.1016/s2213-8587(16)30099-7

21. Moore H, Summerbell C, Greenwood D, et al. Improving management of obesity in primary care: cluster randomised trial. *BMJ*. 2003;327(7423):1085. doi:10.1136/bmj.327.7423.1085

22. Nanchahal K, Power T, Holdsworth E, et al. A pragmatic randomised controlled trial in primary care of the Camden Weight Loss (CAMWEL) programme. *BMJ Open*. 2012;2(3):e000793. doi:10.1136/bmjopen-2011-000793

23. Penn L, White M, Oldroyd J, Walker M, Alberti KG, Mathers JC. Prevention of type 2 diabetes in adults with impaired glucose tolerance: the European Diabetes Prevention RCT in Newcastle upon Tyne, UK. *BMC Public Health*. 2009;9:342. doi:10.1186/1471-2458-9-342

24. Sniehotta FF, Evans EH, Sainsbury K, et al. Behavioural intervention for weight loss maintenance versus standard weight advice in adults with obesity: A randomised controlled trial in the UK (NULevel Trial). *PLoS Med*. 2019;16(5):e1002793. doi:10.1371/journal.pmed.1002793

For peer review only

For peer review only - http://bmjopen.bmj.com/site/about/guidelines.xhtml

25. Astbury NM, Aveyard P, Nickless A, et al. Doctor Referral of Overweight People to Low Energy total diet replacement Treatment (DROPLET): pragmatic randomised controlled trial. *BMJ (Clinical research ed)*. 2018;362:k3760. doi:https://dx.doi.org/10.1136/bmj.k3760

26. Daley A, Jolly K, Madigan C, et al. A brief behavioural intervention to promote regular self-weighing to prevent weight regain after weight loss: a RCT. Published online 2019. doi:https://dx.doi.org/10.3310/phr07070

27. Simpson SA, McNamara R, Shaw C, et al. A feasibility randomised controlled trial of a motivational interviewing-based intervention for weight loss maintenance in adults. *Health Technol Assess*. 2015;19(50):v-vi, xix-xxv, 1-378. doi:10.3310/hta19500

28. Sampson M, Clark A, Bachmann M, et al. Lifestyle Intervention With or Without Lay Volunteers to Prevent Type 2 Diabetes in People With Impaired Fasting Glucose and/or Nondiabetic Hyperglycemia: A Randomized Clinical Trial. *JAMA Internal Medicine*. 2021;181(2):168-178. doi:10.1001/jamainternmed.2020.5938

29. Sterne JAC, Savović J, Page MJ, et al. RoB 2: a revised tool for assessing risk of bias in randomised trials. *BMJ*. 2019;366:l4898.

30. Jonkman NH, Westland H, Trappenburg JCA, et al. Towards tailoring of self-management for patients with chronic heart failure or chronic obstructive pulmonary disease: a protocol for an individual patient data meta-analysis. *BMJ Open*. 2014;4(5):e005220.

31. Oude Hengel KM, Coenen P, Robroek SJW, et al. Socioeconomic inequalities in reach, compliance and effectiveness of lifestyle interventions among workers: protocol for an individual participant data meta-analysis and equity-specific reanalysis. *BMJ Open*. 2019;9(2):e025463. doi:10.1136/bmjopen-2018-025463

32. Hunter KE, Johnson BJ, Askie L, et al. Transforming Obesity Prevention for CHILDren (TOPCHILD) Collaboration: protocol for a systematic review with individual participant data meta-analysis of behavioural interventions for the prevention of early childhood obesity. *BMJ Open*. 2022;12(1):e048166. doi:10.1136/bmjopen-2020-048166

33. Riley RD, Tierney JF, Stewart LA. *Individual Participant Data Meta-Analysis. A Handbook for Healthcare Research*. 1st ed. Wiley; 2021.

34. NHS Digital. Health Survey for England 2019: Overweight and obesity in adults and children. Published 2020. Accessed October 28, 2022. https://files.digital.nhs.uk/9D/4195D5/HSE19-Overweight-obesity-rep.pdf

35. Booth HP, Prevost AT, Gulliford MC. Access to weight reduction interventions for overweight and obese patients in UK primary care: population-based cohort study. *BMJ Open*. 2015;5(1):e006642. doi:10.1136/bmjopen-2014-006642

36. Coulman KD, Redaniel T, Margelyte R, et al. *Patterns of Adult Weight Management Referrals in Primary Care in England*. University of Bristol; 2022.

37. Birch JM, Griffin SJ, Kelly MP, Ahern AL. Inequalities in the uptake of, adherence to and effectiveness of behavioural weight management interventions: systematic review protocol. *BMJ Open*. Published online 2020. doi:10.1136/bmjopen-2020-039518

For peer review only
For peer review only - http://bmjopen.bmj.com/site/about/guidelines.xhtml

1

For peer review only

For peer review only - http://bmjopen.bmj.com/site/about/guidelines.xhtml

Table 1 Summary of eligible studies for inclusion in IPD meta-analysis

Study name (first author)	Year published	Intervention group description	Control group description	Participant eligibility criteria
**WRAP (Ahern)[11]**	2017	Participants were given vouchers to attend WW (previously Weight Watchers) meetings once a week and access WW digital tools for the duration of their intervention (12- or 52-weeks) for free.	A printed booklet of self-help weight-management strategies (British Heart Foundation).	Inclusion: Aged $\geq$18 years and BMI $\geq$28 kg/m^2.<br><br>Exclusion: Planned or current pregnancy, previous or planned bariatric surgery, current participation in a weight-loss programme, having an eating disorder, non-English speaking.
**BeWEL (Anderson)[12]**	2014	12-month intervention delivered by trained lifestyle counsellors in 3 x 1-hour one-to-one visits during the first 3 months, followed by 9 monthly 15-minute telephone conversations, leading to a total contact time of 5.25 hours.	A printed booklet of self-help weight-management strategies (British Heart Foundation).	Inclusion: Aged 50 to 74 years, had undergone polypectomy for adenoma, and BMI >25 kg/m^2.<br><br>Exclusion: Pregnancy, insulin dependent diabetes mellitus, and any cancer diagnosis.
**BWeL (Aveyard)[13]**	2016	General Practitioners offered participants referral to a commercial weight management programme of 12 1-hour sessions (Slimming World, Rosemary Conley) and gave vouchers to allow them to attend for free.	General Practitioners advised participants to lose weight	Inclusion: Aged $\geq$ 18 years, BMI $\geq$25 kg/m^2 (if Asian ethnicity) or BMI $\geq$30 kg/m^2 (if other ethnicities), have a raised body fat percentage.<br><br>Exclusion: Planned or current pregnancy, previous bariatric surgery, completed or participating in a weight management programme within previous 3 months, non-English speaking.
**Ten Top Tips *[10TT]* (Beeken)[14]**	2017	10TT was a self-guided leaflet-based intervention that used habit-formation theory to aid weight loss. A logbook was provided for participants to self-monitor target behaviours.	Usual care, dependent on the participant's General Practitioner. May include dietary advice or referral to a commercial programme.	Inclusion: Aged $\geq$ 18 years, BMI $\geq$ 30 kg/m^2.<br><br>Exclusion: Unable to provide informed consent due to mental incapacity or active psychotic illness, pregnant, or terminally ill.

**PODOSA (Bhopal)[15]**	2014	15 visits from a dietitian over 3 years, where the dietitian would advise participants on achieving weight loss through culturally adapted and translated resources.	4 visits from a dietitian over 3 years where standard advice on healthy eating, diabetes prevention and physical activity was given.	Inclusion: Aged $\geq$ 35 years, self-identified men and women of Indian or Pakistani origin with waists measuring $\geq$ 90cm (men) or $\geq$ 80cm (women), impaired glucose tolerance or impaired fasting glucose tolerance, the family cook was cooperative.<br><br>Exclusion: Receiving long-term oral corticosteroids or weight loss medication, having long-term health disorders making adherence improbable, pregnant, and unlikely to remain in the UK for 3 years.
**Waste the Waist (Greaves)[16]**	2015	4 x 2-hour group-based sessions in the first month to support behaviour change for weight loss, then 5 x 90-minute group sessions over the next 8 months to support maintenance of behaviour change, totalling 13.5 hours of contact time.	Participants were provided written information on the effects of diet and physical activity on cardiovascular risk.	Inclusion: Aged 40-74 years, BMI $\geq$ 28 kg/m^2, and having a high cardiovascular risk defined using either the Framingham or QRISK2 algorithm.<br><br>Exclusion: Existing heart disease, type 2 diabetes mellitus, BMI > 40 kg/m^2,
**Football Fans in Training (Hunt)[17]**	2014	12 weekly sessions of 90-minutes in length, delivered at 13 Scottish professional football club stadiums. Each 90 min session combined advice on healthy diet with physical activity. The balance of classroom and physical activity sessions changed during the 12 weeks; later weeks focused on physical activity as men became fitter, and the shorter classroom sessions focused on revision. The 12-week active phase was followed by a weight maintenance phase with six post-	12 month waiting list to receive the FFIT intervention.	Inclusion: Men, aged 35-65 years, BMI $\geq$ 28 kg/m^2, completed physical activity readiness questionnaire, not taken part in FFIT previously.<br><br>Exclusion: Blood pressure that contraindicated vigorous exercise (systolic ≥160 mm Hg or diastolic ≥100 mm Hg) were excluded from the more intense physical activity programme sessions.

		programme email prompts during 9 months and a group reunion at the club 6 months after the end of the sessions.		
**(Jebb)**[18]	2011	Participants were given vouchers to attend WW (previously Weight Watchers) meetings once a week for 12 months and access digital tools for free.	Advice from their GP and other standard care in line with national treatment guidelines.	Inclusion: Aged ≥ 18 years, BMI 27-35 kg/m^2, and at least one risk factor for obesity related disease (such central adiposity, type 2 diabetes mellitus without inulin treatment, family history of diabetes).<br><br>Exclusion: Achieved weight loss of ≥ 5kg in previous 3 months, history of clinically diagnosed eating disorder, orthopaedic limitations preventing regular physical activity, untreated thyroid disease or more than one change in thyroid treatment in the previous 6 months; receiving treatment with effects on weight or appetite; gastrointestinal disorders; previous surgical procedure for weight loss; major surgery in the previous 3 months; pregnancy or lactation; insulin-treated diabetes; diabetes diagnosis in the previous 6 months; glycated haemoglobin (HbA1c) of at least 75 mmol/mol (9·0%); heart problems in the previous 3 months; uncontrolled hypertension; new prescription drug for a chronic disorder in the previous 3 months or change in dose in the previous 1 month; history or presence of cancer, with the exception of completely resected basal or squamous cell carcinoma if treatment completed 6 months before enrolment or if treatment was stable; or participation in another clinical trial in the previous 30 days.
**Lighten Up (Jolly)**[19]	2011	In addition to 12 vouchers for free entrance to a local leisure centre, participants were randomised to one of 7 intervention groups (all 12 weeks in	12 vouchers for free entrance to a local leisure centre.	Inclusion: Registered with general practices in the South Birmingham Primary Care Trust, aged ≥ 18 years, had a raised BMI recorded in primary care notes within previous 15 months (White Europeans and all ethnic groups apart from South Asians

For peer review only

		length): Weight Watchers, Slimming World, Rosemary Conley, Size Down, GP-led one-to-one counselling, pharmacy-led one-to-one counselling, or a choice of any of the six intervention programmes.		with no comorbidities BMI 30 ≥ kg/m^2, White Europeans and all ethnic groups apart from South Asians with comorbidities BMI 28 ≥ kg/m^2, South Asians with no comorbidities 25 ≥ kg/m^2, South Asians with comorbidities 23 ≥ kg/m^2).<br><br>Exclusion: Unable to understand English or were pregnant.
**POWeR+ (Little)[20]**	2016	Two intervention groups, both consisting of a 24-session web-based weight management programme lasting 6 months.<br><br>POWeR+F provided three scheduled (and four optional) face-to-face nurse support sessions.<br><br>POWeR+R included three phone or email contacts and two optional phone or email contacts.	Brief advice web-pages for a healthier diet.	Inclusion: Aged ≥ 18 years and BMI ≥ 30 kg/m2 or more (or ≥ 28 kg/m2 with hypertension, hypercholesterolaemia, or diabetes).<br><br>Exclusion: Severe mental health problems, too ill to participate in the study or unable to change diet due to health, pregnant or breastfeeding, perceived inability to walk 100m, another member of household participating, no regular access to the internet.
**(Moore)[21]**	2003	Intervention was targeted at general practitioners and practice nurses, and the unit of randomisation was primary care practice. The intervention consisted of 3x90-minute sessions that trained the practitioners on a model approach to obesity treatment.	Control practices were asked to provide usual care to patients.	Inclusion: Aged 16-64 years and BMI ≥ 30 kg/m^2.
**CAMWEL (Nanchahal)[22]**	2012	One-to-one programme delivered across 14 visits over 12 months by advisors trained in obesity causes, diet and physical activity, behaviour change strategies,	Usual care.	Inclusion: Aged ≥ 18 years and BMI ≥25 kg/m^2.

For peer review

		motivational interviewing and cognitive behavioural therapy techniques.		
**EDIPS (Penn)[23]**	2009	Participants received regular individual advice from a dietitian and physiotherapist trained in motivational interviewing. Participants were also invited to group events, such as 'cook and eat'. Individual sessions were for 30 minutes monthly for the first 3 months and then every 3 months for up to 5 years.	Brief advice and usual care from GP.	Inclusion: Aged >40 years, BMI > 25kg/m^2, and impaired glucose tolerance of ≥ 7.8 mmol/l and < 11.1 mmol/l.<br><br>Exclusion: Previous diagnosis of diabetes mellitus, chronic illness that makes participation in moderate physical activity impossible, or on a special diet for medical reasons.
**NULevel (Sniehotta)[24]**<br><br>***Weight loss maintenance***	2019	Intervention was delivered via a combination of a single face-to face meeting and regular text messaged (at least 1 every 2 days). The text messages consisted of content that was triggered by participants daily self-weighing and questionnaire completion.	Brief lifestyle advice received by text message on 4 occasions, 3 months apart.	Inclusion: Aged $\geq$ 18 years, BMI $\geq$ 30kg/m^2 ($\geq$ 28kg/m^2 if of South Asian descent) in the 24 months preceding trial, and had lost $\geq$5% of body weight in the preceding 12 months.<br><br>Exclusion: Lost weight through illness or surgical procedures, pregnant or planning to become pregnant during study period, breastfeeding, unable to understand English, diagnosis of an eating disorder or condition that limits physical activity, or plans to leave geographic area during study period.
**DROPLET (Astbury)[25]**	2018	The intervention was a Total Diet Replacement programme, which consisted of weekly behavioural support for 12 weeks and monthly support for 3 months with formula food products providing 810 kcal per day for the first 8 weeks, followed by gradual reintroduction of food.	Behavioural support for weight loss from a practice nurse and a diet programme with modest energy restriction.	Inclusion: Aged $\geq$ 18 years, BMI $\geq$ 30kg/m^2, and participants' GP determines weight loss would benefit health.<br><br>Exclusion: Scheduled or previously received bariatric surgery, currently participating in a weight management programme, and contraindications to total diet replacement.
**(Daley)[26]**	2019	Over the course of a 12-week weight maintenance programme, participants carried out daily self-weighting, received 3	Brief advice leaflet.	Inclusion: Aged $\geq$ 18 years, had lost $\geq$ 5% of their weight by the end of their weight loss programme, owned a mobile or landline

Weight loss maintenance		brief support phone calls delivered by non-specialist call centre staff, and text messages sent every other day for the first 4 weeks and twice weekly for the remaining 8 weeks.		phone that could receive text messages, was able to understand English sufficiently to complete study procedures.<br><br>Exclusion: Pregnant or intending to become pregnant during the study period.
**WILMA (Simpson)[27]**<br><br>***Weight loss maintenance***	2015	Two intervention groups, both comprising of a 12-month intervention. Participants in both intervention groups could attend 4 peer group support sessions lasting 1.5 hours for 4 months following the face-to-face sessions.<br><br>Intensive group – participants received 6 one-to-one face-to-face individually tailored motivational interviewing delivered fortnightly for 3 months (each session listing around 60 minutes). In the remaining 9 months of the intervention, participants received monthly motivational interviewing calls lasting around 20 minutes.<br><br>Less intensive group – participants received two face-to-face motivational interviewing sessions two weeks apart and two motivational interviewing phone calls at 6-months and 12-months.	Brief advice leaflet.	Inclusion: Aged 18-70 years, current or previous BMI of $\geq$ 30 kg/m^2, and intentionally lost $\geq$5% of body weight in previous 12 months.<br><br>Exclusion: Previous bariatric surgery (unless reversed), terminal illness, inability to understand study materials in English, living with another study participant, or currently pregnant.

**Norfolk Diabetes Prevention Study**[28]	2021	Two intervention groups: Lifestyle intervention and Lifestyle intervention plus diabetes prevention mentors.<br><br>Lifestyle intervention group – delivered by healthcare professionals, involved 6 x 2-hour group sessions to promote behaviour change for weight loss, followed by up to 15 maintenance sessions over the next 46 months. Maximum contact time was 49.5 hours.<br><br>Lifestyle intervention plus diabetes prevention mentors – same as the Lifestyle intervention group with the addition of individual motivational telephone calls between sessions with trained volunteers who had type 2 diabetes.	Usual care consisting of written information and discussion about the risk of diabetes and the effect of lifestyle modification on reducing this risk, delivered by a diabetes prevention facilitator.	Inclusion: Aged $\geq$ 40 years, BMI of $\geq$30kg/m^2, be of a high-risk glycaemic category (either elevated fasting plasma glucose level alone ($\geq$110 and <126 mg/dL) or elevated glycated haemoglobin level ($\geq$6.0% to <6.5%) with an elevated fasting plasma glucose level ($\geq$100 to <110 mh/dL).<br><br>Exclusion: Pregnant or lactating, self-reported conditions which could affect trial results or patient clinical wellbeing such as terminal illness, antipsychotic medication or high dose oral steroids, taking part in any research study involving dietary or lifestyle change intervention.

For peer review only

BMJ Open

Are there inequalities in the attendance and effectiveness of behavioural weight management interventions for adults in the UK? Protocol for an individual participant data (IPD) meta-analysis

Journal:	*BMJ Open*
Manuscript ID	bmjopen-2022-067607.R1
Article Type:	Protocol
Date Submitted by the Author:	18-Jan-2023
Complete List of Authors:	Birch, Jack; MRC Epidemiology Unit<br>Mueller, Julia; University of Cambridge, MRC Epidemiology unit<br>Sharp, Stephen; University of Cambridge, MRC Epidemiology Unit<br>Logue, Jennifer; Lancaster University, Lancaster Medical School<br>Kelly, Michael; University of Cambridge Department of Public Health and Primary Care, Public Health and Primary Care<br>Griffin, Simon; MRC Epidemiology Unit; University of Cambridge Department of Public Health and Primary Care<br>Ahern, Amy; MRC Epidemiology Unit, University of Cambridge
**Primary Subject Heading**:	Public health
Secondary Subject Heading:	Health services research
Keywords:	PUBLIC HEALTH, PRIMARY CARE, PREVENTIVE MEDICINE

SCHOLARONE™
Manuscripts

Are there inequalities in the attendance and effectiveness of behavioural weight management interventions for adults in the UK? Protocol for an individual participant data (IPD) meta-analysis

Authors: Jack M Birch[1], Julia Mueller[1], Stephen J Sharp[1], Jennifer Logue[2], Michael P Kelly[3], Simon J Griffin[1,3], Amy L Ahern[1]

Corresponding author

Jack M Birch

MRC Epidemiology Unit, University of Cambridge School of Clinical Medicine, Box 285 Institute of Metabolic Science, Cambridge Biomedical Campus, CB2 0QQ

jack.birch@mrc-epid.cam.ac.uk

Affiliations: [1]MRC Epidemiology Unit, University of Cambridge; [2]Lancaster Medical School, University of Lancaster [3]Department of Public Health and Primary Care, University of Cambridge.

Key words: obesity, inequalities, individual participant data meta-analysis, weight loss

Word count: 3661

For peer review only

For peer review only - http://bmjopen.bmj.com/site/about/guidelines.xhtml

Abstract

Introduction: It is important to identify whether behavioural weight management interventions work well across different groups in the population so health inequalities in obesity are not widened. Previous systematic reviews of inequalities in the attendance and effectiveness of behavioural weight management interventions have been limited because few trials report relevant analyses and heterogeneity in the categorisation of inequality characteristics prevents meta-analysis. An individual participant data meta-analysis (IPD-MA) allows us to re-analyse all trials with available data in a uniform way. We aim to conduct an IPD meta-analysis of UK randomised controlled trials to examine whether there are inequalities in the attendance and effectiveness of behavioural weight interventions.

Methods and analysis: In a recently published systematic review, we identified 18 UK-based randomised controlled trials of primary care-relevant behavioural interventions, conducted in adults living with overweight or obesity, and reporting weight outcomes at baseline and 1-year follow up. The corresponding author of each trial will be invited to contribute data to the IPD-MA. The outcomes of interest are weight change between baseline and 1-year, and intervention attendance (number of sessions offered versus number of sessions attended). We will primarily consider whether there is an interaction between intervention group and characteristics where inequalities occur, such as by gender/sex, socioeconomic status, or age. The IPD-MA will be conducted in line with the Preferred Reporting Items for Systematic Review and Meta-analysis of IPD (PRISMA-IPD) guidelines.

Ethics and dissemination: No further ethical approval was required as ethical approval for each individual study was obtained by the original trial investigators from appropriate ethics committees. The completed IPD-MA will be disseminated at conferences, in a peer-reviewed journal, and contribute to the lead author's PhD thesis. Investigators of each individual study included in the final IPD-MA will be invited to collaborate on any publications that arise from the project.

For peer review only

For peer review only - http://bmjopen.bmj.com/site/about/guidelines.xhtml

Strengths and limitations of this study

- To the best of our knowledge, this will be the first individual participant data meta-analysis examining inequalities in the attendance and effectiveness of behavioural weight management interventions.
- Individual participant data meta-analyses allow for data harmonisation, which increases the amount of data that can be used and the statistical power to explore whether inequalities are present in the attendance and outcome of behavioural weight management interventions.
- The findings of this study may identify groups that these interventions are less effective in, informing future public health research and practice.
- Due to complexities in developing data sharing agreements and harmonisation of inequality variables in acquiring data from other countries, only UK-based trials are included in this individual participant data meta-analysis.

For peer review only

For peer review only - http://bmjopen.bmj.com/site/about/guidelines.xhtml

1. Introduction

Inequalities in overweight and obesity are widely recognised – those who experience higher levels of socioeconomic deprivation, especially women, are more likely to live with obesity than those who more affluent.[1,2] Similarly, comorbidities of obesity are more common in those experiencing socioeconomic deprivation.[3] It has also been suggested that interventions focusing on individual behaviour change, such as behavioural weight management interventions, may exacerbate health inequalities.[4,5] These intervention-generated inequalities may occur at different stages, including intervention uptake, attendance and effectiveness, and across many individual characteristics that stratify health opportunities (such as access to healthcare) and outcomes. These characteristics are summarised by the PROGRESS-Plus framework: Place of Residence, Race/ethnicity, Occupation, Gender/sex, Education, Socioeconomic status, Social Capital, plus other factors for which discrimination could occur such as age and sexual orientation.[6]

We recently conducted a systematic review to synthesise evidence on how different measures of inequality moderate the uptake, attendance and effectiveness of behavioural weight management interventions in adults.[7] We found that most trials did not consider whether inequalities were generated in the studied intervention; where these analyses were conducted, most found no evidence of inequalities. Where an inequalities gradient was observed, intervention uptake, adherence and attrition generally favoured those considered as 'more advantaged' (such as those who are White, with higher income, or older). Due to substantial differences in the reporting of measures of inequality, together with the low level of reporting of analyses of inequalities, we were unable to perform a quantitative synthesis of the reported results. Hence, it is not possible to fully explore inequalities using aggregated data from published literature alone. This lack of reporting may have occurred because individual trials may not be large enough to detect an interaction between moderators such as socioeconomic status [SES] and the outcome; they are likely just to have been designed to detect an overall effect.

For peer review only - http://bmjopen.bmj.com/site/about/guidelines.xhtml

These limitations can be addressed in part by conducting a meta-analysis of individual participant data (IPD), which requires the central collation, aggregation and reanalysis of IPD from relevant trials.[8,9] This allows for data in each study to be analysed and defined in a uniform way, overcoming heterogeneity issues associated with using aggregate data. Meta-analysis of IPD may provide sufficient statistical power to consider whether there are inequalities in uptake, attendance and effectiveness of interventions.[8,10]

1.2 Research questions

1. To what extent does the effectiveness of behavioural weight management interventions (defined as the difference in weight change between intervention and control) differ by individual characteristics that stratify health opportunities and outcomes (as defined using the PROGRESS-Plus Framework)?
2. To what extent do the weight outcomes of those who have participated in a behavioural weight management trial (defined as weight change in the overall cohort) differ by individual characteristics that stratify health opportunities and outcomes?
3. To what extent does attendance at behavioural weight management interventions differ by individual characteristics that stratify health opportunities and outcomes?

2. Methods and analysis

This IPD meta-analysis responds to limitations identified in our previous systematic review on inequalities in the uptake of, adherence to, and effectiveness of behavioural weight management interventions. The Preferred Reporting Items for Systematic Reviews and Meta-Analyses IPD extension will be followed when reporting this study.[11]

For peer review only - http://bmjopen.bmj.com/site/about/guidelines.xhtml

2.1 Trial identification

2.1.1 Search strategy

This study includes UK-based trials of behavioural weight management interventions that we identified through a previous systematic review.[7] We focused on UK-based trials to reduce heterogeneity in measures of the PROGRESS-Plus characteristics and in the context in which the interventions were delivered. Characteristics such as ethnicity and socioeconomic status are conceptualised differently in different countries which makes synthesising data across these characteristics inappropriate or not possible. For example, socioeconomic status in the UK is often captured using Indices of Multiple Deprivation (IMD), an area-based measure, which is not replicated in other countries. There are also pragmatic reasons for focusing on UK-based trials; the complexity of arranging cross-country data sharing would have made the timelines for this project unviable.

Studies published since the search strategy in the systematic review was conducted, were identified through an updated Medline search and through discussions with the corresponding authors of the included trials. The inclusion and exclusion criteria we used to identify relevant trials for this IPD meta-analysis are:

1. Participants: adults aged 18 years and over with overweight or obesity (body mass index [BMI] > 25 kg/m2 with no upper limit) who were deemed suitable (either by the applicable study team or healthcare practitioner) for weight loss or weight loss maintenance intervention. Participants may have additional risk factors such as hypertension, dyslipidaemia, impaired glucose tolerance or impaired fasting glucose. Studies were excluded if the population was not selected based on a weight-related measure, included participants with BMI<25 kg/m2, were selected based on having a chronic disease where weight loss is part of disease management or being pregnant, or if the intervention was targeted at parents to change behaviour of children. Only studies conducted in the UK were eligible.

For peer review only - http://bmjopen.bmj.com/site/about/guidelines.xhtml

2. Interventions: behavioural weight management interventions with the primary aim of supporting weight loss or weight loss maintenance. Studies were included if they were conducted in, or were applicable to, primary care settings. Interventions may have been delivered alone or as part of a wider multicomponent intervention targeting diet and nutrition, physical activity, sedentary behaviour, or a combination of these. Studies of pharmacological and surgical interventions were excluded unless the trial included behavioural only and control arms. Interventions were considered feasible for application to primary care if they were conducted in a healthcare setting or were widely available in the community at a national or regional level (such as commercial weight loss programmes, text-message and other digital-based interventions); examples of settings that are not relevant to primary care include interventions delivered in inpatient settings, or in residential care homes.
3. Comparators: wait-list control, usual care, or minimal intervention (such as generic print or electronic materials).
4. Outcomes: studies must report weight change in kg at a time point between the 12- or 18-month follow up.
5. Study designs: randomised or cluster-randomised controlled trials [RCTs].

The trials we identified as being eligible for inclusion are outlined in Supplementary Table 1.[12–28]

2.2 Study outcomes, exposures and covariates

2.2.1 Outcomes

Outcomes are weight (kg) at 12-month follow up and intervention attendance. Where data allows, attendance will be measured as the percentage of offered sessions which were attended.

For peer review only - http://bmjopen.bmj.com/site/about/guidelines.xhtml

2.2.2 Exposures

Exposure variables are selected measures of the PROGRESS-Plus criteria where data are likely to be available in UK-based trials of behavioural weight management interventions. Coding of each exposure variable will depend on the variables and coding used in each study providing individual participant data. Should the provided variables be suitably homogeneous, then we anticipate that the coding for the IPD meta-analysis will consist of the following:

- Ethnicity (coded using the England and Wales census categories: Asian or Asian British/Black, African, Caribbean or Black British/Mixed or Multiple ethnic groups/Other ethnic group/White/Missing)
- Occupation (Employed/Unemployed/Retired/Other (i.e. student, carer, voluntary work)/Missing)
- Gender/sex (Male/Female/Non-Binary/Not provided or other)
- Religion (coded using the England and Wales census categories: No religion/Christian/Buddhist/Hindu/Jewish/Muslim/Sikh/Other religion/Missing)
- Education (University degree or equivalent, or higher/Post-secondary education/A-levels or equivalent/GCSEs or equivalent/No formal qualifications/Missing)
- SES – Household income (<£40 000/$\geq$£40 000/Missing)
- SES – Indices of Multiple Deprivation [IMD] (1 –most deprived/2/3/4/5 – least deprived/Missing)
- Social capital – Relationship status (Single/Married or co-habiting/Widowed, separated or divorced/Missing)
- Age (Continuous integer values)
- Disability (Has physical- or mental health-related disability/No disability/Missing)
- Randomised group (Control/Intervention)

For peer review only

2.2.3 Covariates

- Baseline weight (kg)

2.3 Risk of bias assessment

We will use Cochrane's risk of bias tool for randomised controlled trials (RoB 2) to assess the risk of bias in all studies meeting our inclusion criteria.[29] The tool facilitates researchers to consider bias across six domains: the randomisation process; allocation concealment; participant and trial personnel blinding; blinding of outcome assessment; incomplete outcome data; and selective reporting. A rating of 'low risk', 'high risk', or 'unclear' will be assigned to each domain by two contributors independently. Where disagreements occur, these will be resolved by discussion to reach consensus or through consultation with a third contributor. We will present the results of the risk of bias assessment in a summary figure outlining a study's overall risk of bias in addition to the risk of bias in each domain.

2.4 Data collection and management

Our approach to collecting and aggregating the individual participant data was informed by the PRISMA-IPD extension and previously published IPD meta-analysis protocols.[8,11,30–32]

2.4.1 Invitation of authors

Trial investigators of all eligible trials were invited by email, using contact details acquired through trial publications, to contribute data and collaborate on this study. The email outlined our research aims and the specific data we were requesting.

2.4.2 Data collection

Standardised data specification forms will be sent to trial authors. Data will be requested in Microsoft Excel format; however, data will be accepted in any format. Once received, a master copy of each trial

For peer review only - http://bmjopen.bmj.com/site/about/guidelines.xhtml

dataset will be saved in its original format and preserved. Any non-Microsoft Excel format datasets will be converted and then imported into Stata version 16 (StataCorp. 2019. Stata Statistical Software: Release 16.). We will also ask for detailed definitions of the measures used in the trial so we can ensure appropriate harmonisation.

2.4.3 Data checking

Once data are received from trial authors, they will be checked for quality and to ensure they pertain to the correct trial. Descriptive statistics (sample size, demographic variables, weight loss or BMI change) will be performed for each individual trial; should discrepancies occur between our analysis and the original trial publication, then the study authors will be contacted for clarification. Should clarification not be received, then the size of the discrepancy will be considered. If it is small and unlikely to bias the results, then the data will be included in the IPD meta-analysis. Large data inaccuracy and excessive missing data (versus what is reported in the trial publication) may lead to a trial being excluded.

2.4.3 Database creation and aggregation

A single database will be created containing data from all the trials. There is likely to be differences between studies in the coding of measures of inequalities. For example, there is variation in measures of SES that are used. Hence, once all data are received and the differences in coding across the measures become apparent, we will discuss amongst the core research team (JMB, MPK, SJG, and ALA) the best approach to achieve consistent reporting across all measures.

Once the data checking has been completed, variables will be recoded to match the coding of the IPD database. The data from each individual trial will then be copied into the IPD database and checked to ensure the integrity of the data has been maintained through the merge. Each individual trial dataset will be given a unique identifier prior to the merge.

For peer review only

For peer review only - http://bmjopen.bmj.com/site/about/guidelines.xhtml

2.4.4 Trials where IPD are not available

Where we are unable to obtain IPD for an eligible trial, we will ask the trial investigator if they are able to conduct the analyses using the same coding of variables as defined in section 2.5.2 and provide us with the outcome statistics. We will offer this as an option to ensure that we are able to include as much relevant data as possible and we will provide the relevant code to facilitate this. If the outcome statistics are provided from any trials, we will meta-analyse this together with the trials for which we obtained individual participant data. If we synthesise results from data that we did not receive individual participant data for, we will conduct sensitivity analyses excluding these data. Further sensitivity analyses excluding studies with 'high' risk of bias will also be conducted.

2.5 Statistical analysis

Due to our research questions exploring treatment effect and covariate interactions, we decided that a two-stage IPD meta-analysis would be most appropriate. In the first stage, regression analyses are performed individually in each trial. Then in the second stage combines the outcome estimates from each model using a more standard meta-analysis approach (e.g. random-effects meta-analysis).[33] We are using a two-stage approach because it inherently avoids aggregation bias and controls for trial-level confounding, to which one-stage IPD meta-analyses are more susceptible.[33] An additional benefit of performing a two-stage IPD meta-analysis is that trials for which we are unable to acquire individual-level data may still be included in the synthesis provided the relevant outcome statistics can be obtained. Data analysis will be conducted using Stata v16 (StataCorp. 2019. Stata Statistical Software: Release 16. College Station, TX: StataCorp LLC.). The IPD meta-analysis will be performed using the ipdmetan package available in Stata v16. When meta-analysing outcome statistics from trials we received IPD for and trials where these statistics were provided to us, we will use other meta-analysis commands within STATA version 16 to complete these analyses.

For peer review only - http://bmjopen.bmj.com/site/about/guidelines.xhtml

2.5.1 Baseline characteristics

We will describe the baseline characteristics for randomised group and each PROGRESS-Plus characteristic. This will be completed for each trial and as an overall aggregate of all participants included in the meta-analysis. We will compare these characteristics descriptively with data on the prevalence of obesity in the population, such as the Health Survey for England and other studies that have considered who routinely accesses behavioural weight management interventions.[34–36]

2.5.2 Analysis of study outcomes

We will conduct six sets of analyses (if there are sufficient data for each outcome), two for each of our research questions, as we will synthesise data on weight loss interventions separately to data for weight loss maintenance interventions. As all outcomes of interest are continuous, we will use multivariable linear regression models, and include the relevant parameter estimates and standard errors from the models in random-effects meta-analyses. Heterogeneity will be assessed using tau-squared, which summarises between-studies variance, and a 95% prediction interval which indicates the range in which 95% of the true effects lie. Inconsistency will be assessed using I-squared, which indicates the proportion of total variability in the observed effects that is due to heterogeneity.

The subgroups used for each exposure variable are listed below (reference subgroup in bold). If free-text responses are available for any 'other' subgroup for each exposure, we will recode to the most appropriate subgroup in that exposure. If this is not possible, we will recode 'other' to missing. 'Prefer not to say' responses will also be recoded to missing. We anticipate that certain subgroups of some variables will likely have few, if any, data – in particular some sub-categories of religion or relationship status. These will be recoded to missing and excluded from the analyses.

- Ethnicity (**White**/Ethnic minorities (excluding White minorities))
- Occupation (Unemployed/**Employed**/Unable to work/Retired/Student)
- Gender/sex (**Female**/Male/Other or Non-binary)
- Religion (**No religion**/Christian/Buddhist/Hindu/Jewish/Muslim/Sikh/Other religion)

For peer review only - http://bmjopen.bmj.com/site/about/guidelines.xhtml

- Education (No formal qualifications/**GCSEs, O-Levels, or equivalent**/A-Levels or equivalent/Some additional training/University degree)
- Index of Multiple Deprivation (**1 – Most deprived**/2/3/4/5 – Least deprived)
- Household income (**<£40,000**/≥£40,000)
- Relationship status (**Single**/Married or co-habiting/Widowed, separated, or divorced)
- Age (continuous)
- Randomised group (**Control**/Intervention)

We will present summary statistics for weight and attendance outcomes separately for each trial and as combined values across all trials.

2.5.2.1 Research question 1: To what extent does the effectiveness of behavioural weight management interventions (defined as the difference in weight change between intervention and control) differ by individual characteristics that stratify health opportunities and outcomes (as defined using the PROGRESS-Plus Framework)?

These analyses will focus on intervention effects by subgroup. Multivariable linear regression models will be used to test the null hypothesis that there is no interaction between each PROGRESS-Plus characteristic and intervention group on weight at 12 months. Each model will be adjusted for age and gender/sex (with the exception of the models where age and gender/sex are considered as the exposure variables) and baseline weight. The intervention effects will be estimated by subgroup within each trial. These subgroup-specific effects will then be meta-analysed across trials.

2.5.2.2 Research question 2: To what extent do the weight outcomes of those who have participated in a behavioural weight management trial (defined as weight change in the overall cohort) differ by individual characteristics that stratify health opportunities and outcomes?

In these analyses, each trial will be analysed as a cohort study. Using multivariable regression, we will estimate the association between the 'exposure' variable (i.e. each PROGRESS-Plus characteristic we have sufficient data for), and weight at 12-month follow up. Each model will be adjusted for age and gender/sex (with the exception of the models where age and gender/sex are considered as the

For peer review only - http://bmjopen.bmj.com/site/about/guidelines.xhtml

exposure variables), baseline weight, and assigned intervention. Associations will be estimated for each exposure subgroup within a trial, and these associations will then be meta-analysed across trials.

2.5.2.3 Research question 3: To what extent does attendance of behavioural weight management interventions differ by individual characteristics that stratify health opportunities and outcomes?

For the third research question, each trial will be analysed as a cohort study. Attendance will be considered as a percentage – the number of sessions attended divided by the maximum possible number of sessions a participant could attend – and treated as a continuous variable. Multivariable regression models will be used to estimate the association between each PROGRESS-Plus characteristic and attendance. Each model will be adjusted for age and gender/sex (with the exception of the models where age and gender/sex are considered as the exposure variables), and baseline weight, and assigned intervention. Associations will be estimated for each exposure subgroup within a trial, and these associations will then be meta-analysed across trials.

2.5.6 Missing data

A complete-case analysis will be performed, i.e. participants who have missing data for either the outcome, exposure or covariates will be excluded.

2.5.7 Sensitivity analysis

As highlighted in 2.4.4, if we synthesise results from data that we did not receive individual participant data for, we will conduct sensitivity analyses excluding these data. Sensitivity analyses excluding studies with 'high' risk of bias will also be conducted to consider whether these studies have an impact on the results. Should sufficient data be obtained, we will also conduct further analyses to consider whether intervention characteristics affect inequalities in attendance and effectiveness. These

For peer review only

analyses may include comparisons by intervention length, digital versus non-digital, and group based versus individually based.

2.6 Patient and public involvement

As part of the protocol development for the preceding systematic review, we received comments on a lay summary from a patient and public involvement (PPI) representative on the project aims and our definition of the PROGRESS-Plus characteristics.[37] These aims and definitions have been brought forward into this IPD meta-analysis project. We will seek further PPI input on our harmonisation of the subgroups of the exposure variables to ensure our categorisations are appropriate, and a PPI representative will contribute to the interpretation of data and will co-author the final manuscript.

3. Discussion

There is some evidence from our previously conducted systematic review that those we may consider to be 'more advantaged' (such as having more years of education, higher income, being White, and being older) may be the most likely to maintain attendance to and have better outcomes from behavioural weight management interventions.[7] However, evidence was mixed and in that review we were unable to quantitatively synthesise data on attendance and weight outcomes due to heterogeneity in study populations (our review focused on all Organisation for Economic Co-operation and Development countries) and measures of the PROGRESS-Plus characteristics (for example, race and ethnicity are captured very differently in the US versus the UK). This heterogeneity will be partly addressed in this IPD meta-analysis by focusing on trials from a single country (the UK) and through the data harmonisation that can be achieved when access to IPD is obtained.

This IPD meta-analysis will have several implications for public health policy, practice and research. The analyses may identify certain sociodemographic groups that have lesser attendance, or attain lesser weight loss. From a research perspective, future work could seek to establish why this may be

For peer review only - http://bmjopen.bmj.com/site/about/guidelines.xhtml

the case; for public health policy, it is important to identify groups where interventions may be generating or exacerbating inequalities so additional support or provision can be offered to prevent this from occurring.

3.1 Strengths and limitations

There are several strengths of conducting an IPD meta-analysis in comparison to a conventional meta-analysis. IPD meta-analyses are particularly useful for considering moderators of intervention outcomes,[8] due to the increased statistical power gained by pooling data (although this is not guaranteed for all moderating variables, as it depends on the available data). Harmonisation of variables across studies means more data can be pooled together, leading to more robust analyses and conclusions. A further strength of IPD meta-analyses is that they go beyond published data, which may be limited in the measures reported. Receiving the original trial data also allows for increased data checking and increased validation of previously published results.[9]

However, there are also limitations of conducting an IPD meta-analysis. Even though the raw trial data will be acquired, analysis is dependent on the measures assessed in each original trial, and may be limited. Data harmonisation that is required to conduct an IPD meta-analysis may lead to some data being excluded from the analyses as it is unlikely to be possible to harmonise all data from different measures of each PROGRESS-Plus characteristic. A further limitation is that we are only looking at UK-based trials of behavioural weight management interventions, which may limit the generalisability of our findings to other countries or healthcare systems.

3.2 Ethics and dissemination

Ethical approval was not required for this study as no primary data are to be collected, and the IPD are to be analysed in accordance to the purpose for which they were originally collected for. Ethical approval for each eligible trial for this IPD meta-analysis was obtained by the original investigators of each trial.

For peer review only - http://bmjopen.bmj.com/site/about/guidelines.xhtml

We anticipate that the completed IPD meta-analysis will be published in a scientific journal; one collaborator from each trial contributing IPD will be invited to be a co-author on the publication. The findings from this IPD meta-analysis study may also be presented at relevant public health and obesity research conferences, and will contribute to the lead investigator's PhD thesis.

Contributors:

JMB conceived and designed the study, developed the analysis plan, and drafted the manuscript. JM contributed to study design and the analysis plan, and reviewed drafts of the manuscript. SJS contributed to the analysis plan and reviewed drafts of the manuscript. JL, MPK and SJG contributed to study design and reviewed drafts of the manuscript. ALA conceived and designed the study, contributed to the analysis plan, and reviewed drafts of the manuscript. All authors approved the final version for publication.

Funding:

JMB, ALA, SJG, and SJS are supported by the Medical Research Council (MRC) (Grant MC_UU_00006/6). The University of Cambridge has received salary support in respect of SJG from the National Health Service in the East of England through the Clinical Academic Reserve. This work is funded by UKRI grant MC_UU_00006/6.

Conflicts of interest:

JM is a trustee for the Association for the Study of Obesity (unpaid role). JL has received research support from Slimming World and consulting fees from Novo Nordisk and is an employee of AstraZeneca. This work was performed before she became an AstraZeneca employee and AstraZeneca had no role in the work. MPK has undertaken consultancy for Slimming World and led the obesity and weight management guidelines development for NICE from 2005 until 2014. SJG is principal

For peer review only

investigator on a publicly funded (NIHR) trial in which the intervention is provided by WW (formerly Weight Watchers) at no cost. ALA is principal investigator on two publicly funded (NIHR, MRC) trials where the intervention is provided by WW (formerly Weight Watchers) at no cost.

References

1. Newton S, Braithwaite D, Akinyemiju TF. Socio-economic status over the life course and obesity: Systematic review and meta-analysis. *PloS one*. 2017;12(5):e0177151-e0177151.

2. El-Sayed AM, Scarborough P, Galea S. Unevenly distributed: a systematic review of the health literature about socioeconomic inequalities in adult obesity in the United Kingdom. *BMC public health*. 2012;12(1):18.

3. Khanolkar AR, Patalay P. Socioeconomic inequalities in co-morbidity of overweight, obesity and mental ill-health from adolescence to mid-adulthood in two national birth cohort studies. *The Lancet Regional Health – Europe*. 2021;6. doi:10.1016/j.lanepe.2021.100106

4. White M, Adams J, Heywood P. How and why do interventions that increase health overall widen inequalities within populations? In: Babones SJ, ed. *Social Inequality and Public Health*. Policy Press; 2009. https://books.google.co.uk/books?id=NHRoDwAAQBAJ

5. Adams J, Mytton O, White M, Monsivais P. Why Are Some Population Interventions for Diet and Obesity More Equitable and Effective Than Others? The Role of Individual Agency. *PLOS Medicine*. 2016;13(4):e1001990.

6. O'Neill J, Tabish H, Welch V, et al. Applying an equity lens to interventions: using PROGRESS ensures consideration of socially stratifying factors to illuminate inequities in health. *J Clin Epidemiol*. 2014;67(1):56-64.

7. Birch JM, Jones RA, Mueller J, et al. A systematic review of inequalities in the uptake of, adherence to, and effectiveness of behavioral weight management interventions in adults. *Obesity Reviews*. 2022;23(6):e13438. doi:10.1111/obr.13438

8. Weitz E, Kleiboer A, van Straten A, Hollon SD, Cuijpers P. Individual patient data meta-analysis of combined treatments versus psychotherapy (with or without pill placebo), pharmacotherapy or pill placebo for adult depression: a protocol. *BMJ Open*. 2017;7(2):e013478.

9. Stewart LA, Tierney JF. To IPD or not to IPD? Advantages and disadvantages of systematic reviews using individual patient data. *Eval Health Prof*. 2002;25(1):76-97.

10. Chow CK, Islam SMS, Farmer A, et al. Text2PreventCVD: protocol for a systematic review and individual participant data meta-analysis of text message-based interventions for the prevention of cardiovascular diseases. *BMJ Open*. 2016;6(10):e012723.

11. Stewart LA, Clarke M, Rovers M, et al. Preferred Reporting Items for a Systematic Review and Meta-analysis of Individual Participant Data: The PRISMA-IPD Statement. *Jama*. 2015;313(16):1657-1665.

For peer review only - http://bmjopen.bmj.com/site/about/guidelines.xhtml

12. Ahern AL, Wheeler GM, Aveyard P, et al. Extended and standard duration weight-loss programme referrals for adults in primary care (WRAP): a randomised controlled trial. *Lancet (London, England)*. 2017;389(10085):2214-2225. doi:https://dx.doi.org/10.1016/S0140-6736(17)30647-5

13. Anderson AS, Craigie AM, Caswell S, et al. The impact of a bodyweight and physical activity intervention (BeWEL) initiated through a national colorectal cancer screening programme: randomised controlled trial. *BMJ*. 2014;348:g1823. doi:10.1136/bmj.g1823

14. Aveyard P, Lewis A, Tearne S, et al. Screening and brief intervention for obesity in primary care: a parallel, two-arm, randomised trial. *The Lancet*. 2016;388(10059):2492-2500. doi:10.1016/S0140-6736(16)31893-1

15. Beeken RJ, Leurent B, Vickerstaff V, et al. A brief intervention for weight control based on habit-formation theory delivered through primary care: results from a randomised controlled trial. *International journal of obesity (2005)*. 2017;41(2):246-254. doi:10.1038/ijo.2016.206

16. Bhopal RS, Douglas A, Wallia S, et al. Effect of a lifestyle intervention on weight change in south Asian individuals in the UK at high risk of type 2 diabetes: a family-cluster randomised controlled trial. *Lancet Diabetes Endocrinol*. 2014;2(3):218-227. doi:10.1016/s2213-8587(13)70204-3

17. Greaves C, Gillison F, Stathi A, et al. Waste the waist: a pilot randomised controlled trial of a primary care based intervention to support lifestyle change in people with high cardiovascular risk. *International Journal of Behavioral Nutrition and Physical Activity*. 2015;12(1):1.

18. Hunt K, Wyke S, Gray CM, et al. A gender-sensitised weight loss and healthy living programme for overweight and obese men delivered by Scottish Premier League football clubs (FFIT): a pragmatic randomised controlled trial. *The Lancet*. 2014;383(9924):1211-1221. doi:10.1016/S0140-6736(13)62420-4

19. Jebb SA, Ahern AL, Olson AD, et al. Primary care referral to a commercial provider for weight loss treatment versus standard care: a randomised controlled trial. *Lancet*. 2011;378(9801):1485-1492. doi:10.1016/s0140-6736(11)61344-5

20. Jolly K, Lewis A, Beach J, et al. Comparison of range of commercial or primary care led weight reduction programmes with minimal intervention control for weight loss in obesity: lighten Up randomised controlled trial. *BMJ*. 2011;343:d6500. doi:10.1136/bmj.d6500

21. Little P, Stuart B, Hobbs FR, et al. An internet-based intervention with brief nurse support to manage obesity in primary care (POWeR+): a pragmatic, parallel-group, randomised controlled trial. *Lancet Diabetes Endocrinol*. 2016;4(10):821-828. doi:10.1016/s2213-8587(16)30099-7

22. Moore H, Summerbell C, Greenwood D, et al. Improving management of obesity in primary care: cluster randomised trial. *BMJ*. 2003;327(7423):1085. doi:10.1136/bmj.327.7423.1085

23. Nanchahal K, Power T, Holdsworth E, et al. A pragmatic randomised controlled trial in primary care of the Camden Weight Loss (CAMWEL) programme. *BMJ Open*. 2012;2(3):e000793. doi:10.1136/bmjopen-2011-000793

24. Penn L, White M, Oldroyd J, Walker M, Alberti KG, Mathers JC. Prevention of type 2 diabetes in adults with impaired glucose tolerance: the European Diabetes Prevention RCT in Newcastle upon Tyne, UK. *BMC Public Health*. 2009;9:342. doi:10.1186/1471-2458-9-342

For peer review only

25. Sniehotta FF, Evans EH, Sainsbury K, et al. Behavioural intervention for weight loss maintenance versus standard weight advice in adults with obesity: A randomised controlled trial in the UK (NULevel Trial). *PLoS Med*. 2019;16(5):e1002793. doi:10.1371/journal.pmed.1002793

26. Astbury NM, Aveyard P, Nickless A, et al. Doctor Referral of Overweight People to Low Energy total diet replacement Treatment (DROPLET): pragmatic randomised controlled trial. *BMJ (Clinical research ed)*. 2018;362:k3760. doi:https://dx.doi.org/10.1136/bmj.k3760

27. Daley A, Jolly K, Madigan C, et al. A brief behavioural intervention to promote regular self-weighing to prevent weight regain after weight loss: a RCT. Published online 2019. doi:https://dx.doi.org/10.3310/phr07070

28. Simpson SA, McNamara R, Shaw C, et al. A feasibility randomised controlled trial of a motivational interviewing-based intervention for weight loss maintenance in adults. *Health Technol Assess*. 2015;19(50):v-vi, xix-xxv, 1-378. doi:10.3310/hta19500

29. Sterne JAC, Savović J, Page MJ, et al. RoB 2: a revised tool for assessing risk of bias in randomised trials. *BMJ*. 2019;366:l4898.

30. Jonkman NH, Westland H, Trappenburg JCA, et al. Towards tailoring of self-management for patients with chronic heart failure or chronic obstructive pulmonary disease: a protocol for an individual patient data meta-analysis. *BMJ Open*. 2014;4(5):e005220.

31. Oude Hengel KM, Coenen P, Robroek SJW, et al. Socioeconomic inequalities in reach, compliance and effectiveness of lifestyle interventions among workers: protocol for an individual participant data meta-analysis and equity-specific reanalysis. *BMJ Open*. 2019;9(2):e025463. doi:10.1136/bmjopen-2018-025463

32. Hunter KE, Johnson BJ, Askie L, et al. Transforming Obesity Prevention for CHILDren (TOPCHILD) Collaboration: protocol for a systematic review with individual participant data meta-analysis of behavioural interventions for the prevention of early childhood obesity. *BMJ Open*. 2022;12(1):e048166. doi:10.1136/bmjopen-2020-048166

33. Riley RD, Tierney JF, Stewart LA. *Individual Participant Data Meta-Analysis. A Handbook for Healthcare Research*. 1st ed. Wiley; 2021.

34. NHS Digital. Health Survey for England 2019: Overweight and obesity in adults and children. Published 2020. Accessed October 28, 2022. https://files.digital.nhs.uk/9D/4195D5/HSE19-Overweight-obesity-rep.pdf

35. Booth HP, Prevost AT, Gulliford MC. Access to weight reduction interventions for overweight and obese patients in UK primary care: population-based cohort study. *BMJ Open*. 2015;5(1):e006642. doi:10.1136/bmjopen-2014-006642

36. Coulman KD, Redaniel T, Margelyte R, et al. *Patterns of Adult Weight Management Referrals in Primary Care in England*. University of Bristol; 2022.

37. Birch JM, Griffin SJ, Kelly MP, Ahern AL. Inequalities in the uptake of, adherence to and effectiveness of behavioural weight management interventions: systematic review protocol. *BMJ Open*. Published online 2020. doi:10.1136/bmjopen-2020-039518

For peer review only - http://bmjopen.bmj.com/site/about/guidelines.xhtml

1

For peer review only

For peer review only - http://bmjopen.bmj.com/site/about/guidelines.xhtml

Table 1 Summary of eligible studies for inclusion in IPD meta-analysis

Study name (first author)	Year published	Intervention group description	Control group description	Participant eligibility criteria
**WRAP (Ahern)**	2017	Participants were given vouchers to attend WW (previously Weight Watchers) meetings once a week and access WW digital tools for the duration of their intervention (12- or 52-weeks) for free.	A printed booklet of self-help weight-management strategies (British Heart Foundation).	Inclusion: Aged $\geq$18 years and BMI $\geq$28 kg/m^2.<br><br>Exclusion: Planned or current pregnancy, previous or planned bariatric surgery, current participation in a weight-loss programme, having an eating disorder, non-English speaking.
**BeWEL (Anderson)**	2014	12-month intervention delivered by trained lifestyle counsellors in 3 x 1-hour one-to-one visits during the first 3 months, followed by 9 monthly 15-minute telephone conversations, leading to a total contact time of 5.25 hours.	A printed booklet of self-help weight-management strategies (British Heart Foundation).	Inclusion: Aged 50 to 74 years, had undergone polypectomy for adenoma, and BMI >25 kg/m^2.<br><br>Exclusion: Pregnancy, insulin dependent diabetes mellitus, and any cancer diagnosis.
**BWeL (Aveyard)**	2016	General Practitioners offered participants referral to a commercial weight management programme of 12 1-hour sessions (Slimming World, Rosemary Conley) and gave vouchers to allow them to attend for free.	General Practitioners advised participants to lose weight	Inclusion: Aged $\geq$ 18 years, BMI $\geq$25 kg/m^2 (if Asian ethnicity) or BMI $\geq$30 kg/m^2 (if other ethnicities), have a raised body fat percentage.<br><br>Exclusion: Planned or current pregnancy, previous bariatric surgery, completed or participating in a weight management programme within previous 3 months, non-English speaking.
**Ten Top Tips *[10TT]* (Beeken)**	2017	10TT was a self-guided leaflet-based intervention that used habit-formation theory to aid weight loss. A logbook was provided for participants to self-monitor target behaviours.	Usual care, dependent on the participant's General Practitioner. May include dietary advice or referral to a commercial programme.	Inclusion: Aged $\geq$ 18 years, BMI $\geq$ 30 kg/m^2.<br><br>Exclusion: Unable to provide informed consent due to mental incapacity or active psychotic illness, pregnant, or terminally ill.

**PODOSA (Bhopal)**	2014	15 visits from a dietitian over 3 years, where the dietitian would advise participants on achieving weight loss through culturally adapted and translated resources.	4 visits from a dietitian over 3 years where standard advice on healthy eating, diabetes prevention and physical activity was given.	Inclusion: Aged $\geq$ 35 years, self-identified men and women of Indian or Pakistani origin with waists measuring $\geq$ 90cm (men) or $\geq$ 80cm (women), impaired glucose tolerance or impaired fasting glucose tolerance, the family cook was cooperative.<br><br>Exclusion: Receiving long-term oral corticosteroids or weight loss medication, having long-term health disorders making adherence improbable, pregnant, and unlikely to remain in the UK for 3 years.
**Waste the Waist (Greaves)**	2015	4 x 2-hour group-based sessions in the first month to support behaviour change for weight loss, then 5 x 90-minute group sessions over the next 8 months to support maintenance of behaviour change, totalling 13.5 hours of contact time.	Participants were provided written information on the effects of diet and physical activity on cardiovascular risk.	Inclusion: Aged 40-74 years, BMI $\geq$ 28 kg/m^2, and having a high cardiovascular risk defined using either the Framingham or QRISK2 algorithm.<br><br>Exclusion: Existing heart disease, type 2 diabetes mellitus, BMI > 40 kg/m^2,
**Football Fans in Training (Hunt)**	2014	12 weekly sessions of 90-minutes in length, delivered at 13 Scottish professional football club stadiums. Each 90 min session combined advice on healthy diet with physical activity. The balance of classroom and physical activity sessions changed during the 12 weeks; later weeks focused on physical activity as men became fitter, and the shorter classroom sessions focused on revision. The 12-week active phase was followed by a weight maintenance phase with six post-	12 month waiting list to receive the FFIT intervention.	Inclusion: Men, aged 35-65 years, BMI $\geq$ 28 kg/m^2, completed physical activity readiness questionnaire, not taken part in FFIT previously.<br><br>Exclusion: Blood pressure that contraindicated vigorous exercise (systolic ≥160 mm Hg or diastolic ≥100 mm Hg) were excluded from the more intense physical activity programme sessions.

		programme email prompts during 9 months and a group reunion at the club 6 months after the end of the sessions.		
**(Jebb)**	2011	Participants were given vouchers to attend WW (previously Weight Watchers) meetings once a week for 12 months and access digital tools for free.	Advice from their GP and other standard care in line with national treatment guidelines.	Inclusion: Aged > 18 years, BMI 27-35 kg/m^2, and at least one risk factor for obesity related disease (such central adiposity, type 2 diabetes mellitus without inulin treatment, family history of diabetes).<br><br>Exclusion: Achieved weight loss of > 5kg in previous 3 months, history of clinically diagnosed eating disorder, orthopaedic limitations preventing regular physical activity, untreated thyroid disease or more than one change in thyroid treatment in the previous 6 months; receiving treatment with effects on weight or appetite; gastrointestinal disorders; previous surgical procedure for weight loss; major surgery in the previous 3 months; pregnancy or lactation; insulin-treated diabetes; diabetes diagnosis in the previous 6 months; glycated haemoglobin (HbA1c) of at least 75 mmol/mol (9·0%); heart problems in the previous 3 months; uncontrolled hypertension; new prescription drug for a chronic disorder in the previous 3 months or change in dose in the previous 1 month; history or presence of cancer, with the exception of completely resected basal or squamous cell carcinoma if treatment completed 6 months before enrolment or if treatment was stable; or participation in another clinical trial in the previous 30 days.
**Lighten Up (Jolly)**	2011	In addition to 12 vouchers for free entrance to a local leisure centre, participants were randomised to one of 7 intervention groups (all 12 weeks in	12 vouchers for free entrance to a local leisure centre.	Inclusion: Registered with general practices in the South Birmingham Primary Care Trust, aged > 18 years, had a raised BMI recorded in primary care notes within previous 15 months (White Europeans and all ethnic groups apart from South Asians

For peer review only

		length): Weight Watchers, Slimming World, Rosemary Conley, Size Down, GP-led one-to-one counselling, pharmacy-led one-to-one counselling, or a choice of any of the six intervention programmes.		with no comorbidities BMI 30 ≥ kg/m^2, White Europeans and all ethnic groups apart from South Asians with comorbidities BMI 28 ≥ kg/m^2, South Asians with no comorbidities 25 ≥ kg/m^2, South Asians with comorbidities 23 ≥ kg/m^2).<br><br>Exclusion: Unable to understand English or were pregnant.
**POWeR+ (Little)**	2016	Two intervention groups, both consisting of a 24-session web-based weight management programme lasting 6 months.<br><br>POWeR+F provided three scheduled (and four optional) face-to-face nurse support sessions.<br><br>POWeR+R included three phone or email contacts and two optional phone or email contacts.	Brief advice web-pages for a healthier diet.	Inclusion: Aged ≥ 18 years and BMI ≥ 30 kg/m2 or more (or ≥ 28 kg/m2 with hypertension, hypercholesterolaemia, or diabetes).<br><br>Exclusion: Severe mental health problems, too ill to participate in the study or unable to change diet due to health, pregnant or breastfeeding, perceived inability to walk 100m, another member of household participating, no regular access to the internet.
**(Moore)**	2003	Intervention was targeted at general practitioners and practice nurses, and the unit of randomisation was primary care practice. The intervention consisted of 3x90-minute sessions that trained the practitioners on a model approach to obesity treatment.	Control practices were asked to provide usual care to patients.	Inclusion: Aged 16-64 years and BMI ≥ 30 kg/m^2.
**CAMWEL (Nanchahal)**	2012	One-to-one programme delivered across 14 visits over 12 months by advisors trained in obesity causes, diet and physical activity, behaviour change strategies,	Usual care.	Inclusion: Aged ≥ 18 years and BMI ≥25 kg/m^2.

For peer review

		motivational interviewing and cognitive behavioural therapy techniques.		
**EDIPS (Penn)**	2009	Participants received regular individual advice from a dietitian and physiotherapist trained in motivational interviewing. Participants were also invited to group events, such as 'cook and eat'. Individual sessions were for 30 minutes monthly for the first 3 months and then every 3 months for up to 5 years.	Brief advice and usual care from GP.	Inclusion: Aged >40 years, BMI > 25kg/m^2, and impaired glucose tolerance of ≥ 7.8 mmol/l and < 11.1 mmol/l.<br><br>Exclusion: Previous diagnosis of diabetes mellitus, chronic illness that makes participation in moderate physical activity impossible, or on a special diet for medical reasons.
**NULevel (Sniehotta)**<br><br>***Weight loss maintenance***	2019	Intervention was delivered via a combination of a single face-to face meeting and regular text messaged (at least 1 every 2 days). The text messages consisted of content that was triggered by participants daily self-weighing and questionnaire completion.	Brief lifestyle advice received by text message on 4 occasions, 3 months apart.	Inclusion: Aged $\geq$ 18 years, BMI $\geq$ 30kg/m^2 ($\geq$ 28kg/m^2 if of South Asian descent) in the 24 months preceding trial, and had lost $\geq$5% of body weight in the preceding 12 months.<br><br>Exclusion: Lost weight through illness or surgical procedures, pregnant or planning to become pregnant during study period, breastfeeding, unable to understand English, diagnosis of an eating disorder or condition that limits physical activity, or plans to leave geographic area during study period.
**DROPLET (Astbury)**	2018	The intervention was a Total Diet Replacement programme, which consisted of weekly behavioural support for 12 weeks and monthly support for 3 months with formula food products providing 810 kcal per day for the first 8 weeks, followed by gradual reintroduction of food.	Behavioural support for weight loss from a practice nurse and a diet programme with modest energy restriction.	Inclusion: Aged $\geq$ 18 years, BMI $\geq$ 30kg/m^2, and participants' GP determines weight loss would benefit health.<br><br>Exclusion: Scheduled or previously received bariatric surgery, currently participating in a weight management programme, and contraindications to total diet replacement.
**LIMIT (Daley)**	2019	Over the course of a 12-week weight maintenance programme, participants carried out daily self-weighting, received 3	Brief advice leaflet.	Inclusion: Aged $\geq$ 18 years, had lost $\geq$ 5% of their weight by the end of their weight loss programme, owned a mobile or landline

Weight loss maintenance		brief support phone calls delivered by non-specialist call centre staff, and text messages sent every other day for the first 4 weeks and twice weekly for the remaining 8 weeks.		phone that could receive text messages, was able to understand English sufficiently to complete study procedures.<br><br>Exclusion: Pregnant or intending to become pregnant during the study period.
**WILMA (Simpson)**<br><br>***Weight loss maintenance***	2015	Two intervention groups, both comprising of a 12-month intervention. Participants in both intervention groups could attend 4 peer group support sessions lasting 1.5 hours for 4 months following the face-to-face sessions.<br><br>Intensive group – participants received 6 one-to-one face-to-face individually tailored motivational interviewing delivered fortnightly for 3 months (each session listing around 60 minutes). In the remaining 9 months of the intervention, participants received monthly motivational interviewing calls lasting around 20 minutes.<br><br>Less intensive group – participants received two face-to-face motivational interviewing sessions two weeks apart and two motivational interviewing phone calls at 6-months and 12-months.	Brief advice leaflet.	Inclusion: Aged 18-70 years, current or previous BMI of $\geq$ 30 kg/m^2, and intentionally lost $\geq$5% of body weight in previous 12 months.<br><br>Exclusion: Previous bariatric surgery (unless reversed), terminal illness, inability to understand study materials in English, living with another study participant, or currently pregnant.

BMJ Open

Are there inequalities in the attendance and effectiveness of behavioural weight management interventions for adults in the UK? Protocol for an individual participant data (IPD) meta-analysis

Journal:	*BMJ Open*
Manuscript ID	bmjopen-2022-067607.R2
Article Type:	Protocol
Date Submitted by the Author:	14-Feb-2023
Complete List of Authors:	Birch, Jack; MRC Epidemiology Unit<br>Mueller, Julia; University of Cambridge, MRC Epidemiology unit<br>Sharp, Stephen; University of Cambridge, MRC Epidemiology Unit<br>Logue, Jennifer; Lancaster University, Lancaster Medical School<br>Kelly, Michael; University of Cambridge Department of Public Health and Primary Care, Public Health and Primary Care<br>Griffin, Simon; MRC Epidemiology Unit; University of Cambridge Department of Public Health and Primary Care<br>Ahern, Amy; MRC Epidemiology Unit, University of Cambridge
**Primary Subject Heading**:	Public health
Secondary Subject Heading:	Health services research
Keywords:	PUBLIC HEALTH, PRIMARY CARE, PREVENTIVE MEDICINE

SCHOLARONE™
Manuscripts

Are there inequalities in the attendance and effectiveness of behavioural weight management interventions for adults in the UK? Protocol for an individual participant data (IPD) meta-analysis

Authors: Jack M Birch[1], Julia Mueller[1], Stephen J Sharp[1], Jennifer Logue[2], Michael P Kelly[3], Simon J Griffin[1,3], Amy L Ahern[1]

Corresponding author

Jack M Birch

MRC Epidemiology Unit, University of Cambridge School of Clinical Medicine, Box 285 Institute of Metabolic Science, Cambridge Biomedical Campus, CB2 0QQ

jack.birch@mrc-epid.cam.ac.uk

Affiliations: [1]MRC Epidemiology Unit, University of Cambridge; [2]Lancaster Medical School, University of Lancaster [3]Department of Public Health and Primary Care, University of Cambridge.

Key words: obesity, inequalities, individual participant data meta-analysis, weight loss

Word count: 3909

For peer review only

Abstract

Introduction: It is important to identify whether behavioural weight management interventions work well across different groups in the population so health inequalities in obesity are not widened. Previous systematic reviews of inequalities in the attendance and effectiveness of behavioural weight management interventions have been limited because few trials report relevant analyses and heterogeneity in the categorisation of inequality characteristics prevents meta-analysis. An individual participant data meta-analysis (IPD-MA) allows us to re-analyse all trials with available data in a uniform way. We aim to conduct an IPD meta-analysis of UK randomised controlled trials to examine whether there are inequalities in the attendance and effectiveness of behavioural weight interventions.

Methods and analysis: In a recently published systematic review, we identified 18 UK-based randomised controlled trials of primary care-relevant behavioural interventions, conducted in adults living with overweight or obesity, and reporting weight outcomes at baseline and 1-year follow up. The corresponding author of each trial will be invited to contribute data to the IPD-MA. The outcomes of interest are weight change between baseline and 1-year, and intervention attendance (number of sessions offered versus number of sessions attended). We will primarily consider whether there is an interaction between intervention group and characteristics where inequalities occur, such as by gender/sex, socioeconomic status, or age. The IPD-MA will be conducted in line with the Preferred Reporting Items for Systematic Review and Meta-analysis of IPD (PRISMA-IPD) guidelines.

Ethics and dissemination: No further ethical approval was required as ethical approval for each individual study was obtained by the original trial investigators from appropriate ethics committees. The completed IPD-MA will be disseminated at conferences, in a peer-reviewed journal, and contribute to the lead author's PhD thesis. Investigators of each individual study included in the final IPD-MA will be invited to collaborate on any publications that arise from the project.

For peer review only - http://bmjopen.bmj.com/site/about/guidelines.xhtml

Strengths and limitations of this study

- To the best of our knowledge, this will be the first individual participant data meta-analysis examining inequalities in the attendance and effectiveness of behavioural weight management interventions.
- Individual participant data meta-analyses allow for data harmonisation, which increases the amount of data that can be used and the statistical power to explore whether inequalities are present in the attendance and outcome of behavioural weight management interventions.
- Due to complexities in developing data sharing agreements and harmonisation of inequality variables in acquiring data from other countries, only UK-based trials are included in this individual participant data meta-analysis.

For peer review only

For peer review only - http://bmjopen.bmj.com/site/about/guidelines.xhtml

1. Introduction

Inequalities in overweight and obesity are widely recognised – those who experience higher levels of socioeconomic deprivation, especially women, are more likely to live with obesity than those who more affluent.[1,2] Similarly, comorbidities of obesity are more common in those experiencing socioeconomic deprivation.[3] It has also been suggested that interventions focusing on individual behaviour change, such as behavioural weight management interventions, may exacerbate health inequalities.[4,5] These intervention-generated inequalities may occur at different stages, including intervention uptake, attendance and effectiveness, and across many individual characteristics that stratify health opportunities (such as access to healthcare) and outcomes. These characteristics are summarised by the PROGRESS-Plus framework: Place of Residence, Race/ethnicity, Occupation, Gender/sex, Education, Socioeconomic status, Social Capital, plus other factors for which discrimination could occur such as age and sexual orientation.[6]

We recently conducted a systematic review to synthesise evidence on how different measures of inequality moderate the uptake, attendance and effectiveness of behavioural weight management interventions in adults.[7] We found that most trials did not consider whether inequalities were generated in the studied intervention; where these analyses were conducted, most found no evidence of inequalities. Where an inequalities gradient was observed, intervention uptake, adherence and attrition generally favoured those considered as 'more advantaged' (such as those who are White, with higher income, or older). Due to substantial differences in the reporting of measures of inequality, together with the low level of reporting of analyses of inequalities, we were unable to perform a quantitative synthesis of the reported results. Hence, it is not possible to fully explore inequalities using aggregated data from published literature alone. This lack of reporting may have occurred because individual trials may not be large enough to detect an interaction between moderators such as socioeconomic status [SES] and the outcome; they are likely just to have been designed to detect an overall effect.

For peer review only - http://bmjopen.bmj.com/site/about/guidelines.xhtml

These limitations can be addressed in part by conducting a meta-analysis of individual participant data (IPD), which requires the central collation, aggregation and reanalysis of IPD from relevant trials.[8,9] This allows for data in each study to be analysed and defined in a uniform way, overcoming heterogeneity issues associated with using aggregate data. Meta-analysis of IPD may provide sufficient statistical power to consider whether there are inequalities in uptake, attendance and effectiveness of interventions.[8,10]

1.2 Research questions

1. To what extent does the effectiveness of behavioural weight management interventions (defined as the difference in weight change between intervention and control) differ by individual characteristics that stratify health opportunities and outcomes (as defined using the PROGRESS-Plus Framework)?
2. To what extent do the weight outcomes of those who have participated in a behavioural weight management trial (defined as weight change in the overall cohort) differ by individual characteristics that stratify health opportunities and outcomes?
3. To what extent does attendance at behavioural weight management interventions differ by individual characteristics that stratify health opportunities and outcomes?

2. Methods and analysis

This IPD meta-analysis responds to limitations identified in our previous systematic review on inequalities in the uptake of, adherence to, and effectiveness of behavioural weight management interventions. The Preferred Reporting Items for Systematic Reviews and Meta-Analyses IPD extension will be followed when reporting this study.[11]

For peer review only - http://bmjopen.bmj.com/site/about/guidelines.xhtml

2.1 Trial identification

2.1.1 Search strategy

This study includes UK-based trials of behavioural weight management interventions that we identified through a previous systematic review.[7] We focused on UK-based trials to reduce heterogeneity in measures of the PROGRESS-Plus characteristics and in the context in which the interventions were delivered. Characteristics such as ethnicity and socioeconomic status are conceptualised differently in different countries which makes synthesising data across these characteristics inappropriate or not possible. For example, socioeconomic status in the UK is often captured using Indices of Multiple Deprivation (IMD), an area-based measure, which is not replicated in other countries. There are also pragmatic reasons for focusing on UK-based trials; the complexity of arranging cross-country data sharing would have made the timelines for this project unviable.

Studies published since the search strategy in the systematic review was conducted, were identified through an updated Medline search and through discussions with the corresponding authors of the included trials. The inclusion and exclusion criteria we used to identify relevant trials for this IPD meta-analysis are:

1. Participants: adults aged 18 years and over with overweight or obesity (body mass index [BMI] > 25 kg/m2 with no upper limit) who were deemed suitable (either by the applicable study team or healthcare practitioner) for weight loss or weight loss maintenance intervention. Participants may have additional risk factors such as hypertension, dyslipidaemia, impaired glucose tolerance or impaired fasting glucose. Studies were excluded if the population was not selected based on a weight-related measure, included participants with BMI<25 kg/m2, were selected based on having a chronic disease where weight loss is part of disease management or being pregnant, or if the intervention was targeted at parents to change behaviour of children. These exclusions were made to reduce heterogeneity between study

For peer review only - http://bmjopen.bmj.com/site/about/guidelines.xhtml

populations and to be consistent with previous reviews.[7,12] Only studies conducted in the UK were eligible.

2. Interventions: behavioural weight management interventions with the primary aim of supporting weight loss or weight loss maintenance. Studies were included if they were conducted in, or were applicable to, primary care settings. Interventions may have been delivered alone or as part of a wider multicomponent intervention targeting diet and nutrition, physical activity, sedentary behaviour, or a combination of these. Studies of pharmacological and surgical interventions were excluded unless the trial included behavioural only and control arms. Interventions were considered feasible for application to primary care if they were conducted in a healthcare setting or were widely available in the community at a national or regional level (such as commercial weight loss programmes, text-message and other digital-based interventions); examples of settings that are not relevant to primary care include interventions delivered in inpatient settings, or in residential care homes.
3. Comparators: wait-list control, usual care, or minimal intervention (such as generic print or electronic materials).
4. Outcomes: studies must report weight change in kg at a time point between the 12- or 18-month follow up.
5. Study designs: randomised or cluster-randomised controlled trials [RCTs].

The trials we identified as being eligible for inclusion are outlined in Supplementary Table 1.[13–29]

2.2 Study outcomes, exposures and covariates

2.2.1 Outcomes

Outcomes are weight (kg) at 12-month follow up and intervention attendance. Where data allows, attendance will be measured as the percentage of offered sessions which were attended.

For peer review only - http://bmjopen.bmj.com/site/about/guidelines.xhtml

2.2.2 Exposures

Exposure variables are selected measures of the PROGRESS-Plus criteria where data are likely to be available in UK-based trials of behavioural weight management interventions. Coding of each exposure variable will depend on the variables and coding used in each study providing individual participant data. Should the provided variables be suitably homogeneous, then we anticipate that the coding for the IPD meta-analysis will consist of the following:

- Ethnicity (coded using the England and Wales census categories: Asian or Asian British/Black, African, Caribbean or Black British/Mixed or Multiple ethnic groups/Other ethnic group/White/Missing)
- Occupation (Employed/Unemployed/Retired/Other (i.e. student, carer, voluntary work)/Missing)
- Gender/sex (Male/Female/Non-Binary/Not provided or other)
- Religion (coded using the England and Wales census categories: No religion/Christian/Buddhist/Hindu/Jewish/Muslim/Sikh/Other religion/Missing)
- Education (University degree or equivalent, or higher/Post-secondary education/A-levels or equivalent/GCSEs or equivalent/No formal qualifications/Missing)
- SES – Household income (<£40 000/≥£40 000/Missing)
- SES – Indices of Multiple Deprivation [IMD] (1 –most deprived/2/3/4/5 – least deprived/Missing)
- Social capital – Relationship status (Single/Married or co-habiting/Widowed, separated or divorced/Missing)
- Age (Continuous integer values)
- Disability (Has physical- or mental health-related disability/No disability/Missing)
- Randomised group (Control/Intervention)

For peer review only

For peer review only - http://bmjopen.bmj.com/site/about/guidelines.xhtml

2.2.3 Covariates

- Baseline weight (kg)

2.3 Risk of bias assessment

We will use Cochrane's risk of bias tool for randomised controlled trials (RoB 2) to assess the risk of bias in all studies meeting our inclusion criteria.[30] The tool facilitates researchers to consider bias across six domains: the randomisation process; allocation concealment; participant and trial personnel blinding; blinding of outcome assessment; incomplete outcome data; and selective reporting. A rating of 'low risk', 'high risk', or 'unclear' will be assigned to each domain by two contributors independently. Where disagreements occur, these will be resolved by discussion to reach consensus or through consultation with a third contributor. We will present the results of the risk of bias assessment in a summary figure outlining a study's overall risk of bias in addition to the risk of bias in each domain.

2.4 Data collection and management

Our approach to collecting and aggregating the individual participant data was informed by the PRISMA-IPD extension and previously published IPD meta-analysis protocols.[8,11,31–33]

2.4.1 Invitation of authors

Trial investigators of all eligible trials were invited by email, using contact details acquired through trial publications, to contribute data and collaborate on this study. The email outlined our research aims and the specific data we were requesting.

2.4.2 Data collection

Standardised data specification forms will be sent to trial authors. Data will be requested in Microsoft Excel format; however, data will be accepted in any format. Once received, a master copy of each trial

For peer review only - http://bmjopen.bmj.com/site/about/guidelines.xhtml

dataset will be saved in its original format and preserved. Any non-Microsoft Excel format datasets will be converted and then imported into Stata version 17 (StataCorp. 2021. Stata Statistical Software: Release 17.). We will also ask for detailed definitions of the measures used in the trial so we can ensure appropriate harmonisation.

2.4.3 Data checking

Once data are received from trial authors, they will be checked for quality and to ensure they pertain to the correct trial. Descriptive statistics (sample size, demographic variables, weight loss or BMI change) will be performed for each individual trial; should discrepancies occur between our analysis and the original trial publication, then the study authors will be contacted for clarification. Should clarification not be received, then the size of the discrepancy will be considered. If it is small and unlikely to bias the results, then the data will be included in the IPD meta-analysis. Large data inaccuracy and excessive missing data (versus what is reported in the trial publication) may lead to a trial being excluded.

2.4.3 Database creation and aggregation

A single database will be created containing data from all the trials. There is likely to be differences between studies in the coding of measures of inequalities. For example, there is variation in measures of SES that are used. Hence, once all data are received and the differences in coding across the measures become apparent, we will discuss amongst the core research team (JMB, MPK, SJG, and ALA) the best approach to achieve consistent reporting across all measures.

Once the data checking has been completed, variables will be recoded to match the coding of the IPD database. The data from each individual trial will then be copied into the IPD database and checked to ensure the integrity of the data has been maintained through the merge. Each individual trial dataset will be given a unique identifier prior to the merge.

For peer review only - http://bmjopen.bmj.com/site/about/guidelines.xhtml

2.4.4 Trials where IPD are not available

Where we are unable to obtain IPD for an eligible trial, we will ask the trial investigator if they are able to conduct the analyses using the same coding of variables as defined in section 2.5.2 and provide us with the outcome statistics. We will offer this as an option to ensure that we are able to include as much relevant data as possible and we will provide the relevant code to facilitate this. If the outcome statistics are provided from any trials, we will meta-analyse this together with the trials for which we obtained individual participant data. If we synthesise results from data that we did not receive individual participant data for, we will conduct sensitivity analyses excluding these data. Further sensitivity analyses excluding studies with 'high' risk of bias will also be conducted.

2.5 Statistical analysis

Due to our research questions exploring treatment effect and covariate interactions, we decided that a two-stage IPD meta-analysis would be most appropriate. In the first stage, regression analyses are performed individually in each trial. Then in the second stage combines the outcome estimates from each model using a more standard meta-analysis approach (e.g. random-effects meta-analysis).[34] We are using a two-stage approach because it inherently avoids aggregation bias and controls for trial-level confounding, to which one-stage IPD meta-analyses are more susceptible.[34] An additional benefit of performing a two-stage IPD meta-analysis is that trials for which we are unable to acquire individual-level data may still be included in the synthesis provided the relevant outcome statistics can be obtained. Data analysis will be conducted using Stata v17 (StataCorp. 2021. Stata Statistical Software: Release 17. College Station, TX: StataCorp LLC.).

2.5.1 Baseline characteristics

We will describe the baseline characteristics for randomised group and each PROGRESS-Plus characteristic. This will be completed for each trial and as an overall aggregate of all participants included in the meta-analysis. We will compare these characteristics descriptively with data on the

For peer review only - http://bmjopen.bmj.com/site/about/guidelines.xhtml

prevalence of obesity in the population, such as the Health Survey for England and other studies that have considered who routinely accesses behavioural weight management interventions.[35–37]

2.5.2 Analysis of study outcomes

We will conduct six sets of analyses (if there are sufficient data for each outcome), two for each of our research questions, as we will synthesise data on weight loss interventions separately to data for weight loss maintenance interventions. As all outcomes of interest are continuous, we will use multivariable linear regression models, and include the relevant parameter estimates and standard errors from the models in random-effects meta-analyses. Heterogeneity will be assessed using tau-squared, which summarises between-studies variance, and a 95% prediction interval which indicates the range in which 95% of the true effects lie. Inconsistency will be assessed using I-squared, which indicates the proportion of total variability in the observed effects that is due to heterogeneity.

The subgroups used for each exposure variable are listed below (reference subgroup in bold). If free-text responses are available for any 'other' subgroup for each exposure, we will recode to the most appropriate subgroup in that exposure. If this is not possible, we will recode 'other' to missing. 'Prefer not to say' responses will also be recoded to missing. We anticipate that certain subgroups of some variables will likely have few, if any, data – in particular some sub-categories of religion or relationship status. These will be recoded to missing and excluded from the analyses.

- Ethnicity (**White**/Ethnic minorities (excluding White minorities))
- Occupation (Unemployed/**Employed**/Unable to work/Retired/Student)
- Gender/sex (**Female**/Male/Other or Non-binary)
- Religion (**No religion**/Christian/Buddhist/Hindu/Jewish/Muslim/Sikh/Other religion)
- Education (No formal qualifications/**GCSEs, O-Levels, or equivalent**/A-Levels or equivalent/Some additional training/University degree)
- Index of Multiple Deprivation (**1 – Most deprived**/2/3/4/5 – Least deprived)
- Household income (**<£40,000**/≥£40,000)
- Relationship status (**Single**/Married or co-habiting/Widowed, separated, or divorced)
- Age (continuous)

For peer review only - http://bmjopen.bmj.com/site/about/guidelines.xhtml

For peer review only

- Randomised group (**Control**/Intervention)

We will present summary statistics for weight and attendance outcomes separately for each trial and as combined values across all trials.

2.5.2.1 Research question 1: To what extent does the effectiveness of behavioural weight management interventions (defined as the difference in weight change between intervention and control) differ by individual characteristics that stratify health opportunities and outcomes (as defined using the PROGRESS-Plus Framework)?

These analyses will focus on intervention effects by subgroup. Multivariable linear regression models will be used to test the null hypothesis that there is no interaction between each PROGRESS-Plus characteristic and intervention group on weight at 12 months. Each model will be adjusted for age and gender/sex (with the exception of the models where age and gender/sex are considered as the exposure variables) and baseline weight. The interaction terms will then be meta-analysed across trials.

2.5.2.2 Research question 2: To what extent do the weight outcomes of those who have participated in a behavioural weight management trial (defined as weight change in the overall cohort) differ by individual characteristics that stratify health opportunities and outcomes?

In these analyses, each trial will be analysed as a cohort study. Using multivariable regression, we will estimate the association between the 'exposure' variable (i.e. each PROGRESS-Plus characteristic we have sufficient data for), and weight at 12-month follow up. Each model will be adjusted for age and gender/sex (with the exception of the models where age and gender/sex are considered as the exposure variables), baseline weight, and assigned intervention. Associations will be estimated for each exposure subgroup within a trial, and these associations will then be meta-analysed across trials.

For peer review only - http://bmjopen.bmj.com/site/about/guidelines.xhtml

2.5.2.3 Research question 3: To what extent does attendance of behavioural weight management interventions differ by individual characteristics that stratify health opportunities and outcomes?

For the third research question, each trial will be analysed as a cohort study. Attendance will be considered as a percentage – the number of sessions attended divided by the maximum possible number of sessions a participant could attend – and treated as a continuous variable. Multivariable regression models will be used to estimate the association between each PROGRESS-Plus characteristic and attendance. Each model will be adjusted for age and gender/sex (with the exception of the models where age and gender/sex are considered as the exposure variables), and baseline weight, and assigned intervention. Associations will be estimated for each exposure subgroup within a trial, and these associations will then be meta-analysed across trials.

2.5.6 Missing data

A complete-case analysis will be performed, i.e. participants who have missing data for either the outcome, exposure or covariates will be excluded.

2.5.7 Sensitivity analysis

As highlighted in 2.4.4, if we synthesise results from data that we did not receive individual participant data for, we will conduct sensitivity analyses excluding these data. Sensitivity analyses excluding studies with 'high' risk of bias will also be conducted to consider whether these studies have an impact on the results. Should sufficient data be obtained, we will also conduct further analyses to consider whether intervention characteristics affect inequalities in attendance and effectiveness. These analyses may include comparisons by intervention length, digital versus non-digital, and group based versus individually based.

For peer review only - http://bmjopen.bmj.com/site/about/guidelines.xhtml

2.6 Patient and public involvement

As part of the protocol development for the preceding systematic review, we received comments on a lay summary from a patient and public involvement (PPI) representative on the project aims and our definition of the PROGRESS-Plus characteristics.[38] These aims and definitions have been brought forward into this IPD meta-analysis project. We will seek further PPI input on our harmonisation of the subgroups of the exposure variables to ensure our categorisations are appropriate, and a PPI representative will contribute to the interpretation of data and will co-author the final manuscript.

3. Discussion

There is some evidence from our previously conducted systematic review that those we may consider to be 'more advantaged' (such as having more years of education, higher income, being White, and being older) may be the most likely to maintain attendance to and have better outcomes from behavioural weight management interventions.[7] However, evidence was mixed and in that review we were unable to quantitatively synthesise data on attendance and weight outcomes due to heterogeneity in study populations (our review focused on all Organisation for Economic Co-operation and Development countries) and measures of the PROGRESS-Plus characteristics (for example, race and ethnicity are captured very differently in the US versus the UK). This heterogeneity will be partly addressed in this IPD meta-analysis by focusing on trials from a single country (the UK) and through the data harmonisation that can be achieved when access to IPD is obtained.

This IPD meta-analysis will have several implications for public health policy, practice and research. The analyses may identify certain sociodemographic groups that have lesser attendance, or attain lesser weight loss. From a research perspective, future work could seek to establish why this may be the case; for public health policy, it is important to identify groups where interventions may be

For peer review only

generating or exacerbating inequalities so additional support or provision can be offered to prevent this from occurring.

3.1 Strengths and limitations

There are several strengths of conducting an IPD meta-analysis in comparison to a conventional meta-analysis. IPD meta-analyses are particularly useful for considering moderators of intervention outcomes,[8] due to the increased statistical power gained by pooling data (although this is not guaranteed for all moderating variables, as it depends on the available data). Harmonisation of variables across studies means more data can be pooled together, leading to more robust analyses and conclusions. A further strength of IPD meta-analyses is that they go beyond published data, which may be limited in the measures reported. Receiving the original trial data also allows for increased data checking and increased validation of previously published results.[9]

However, there are also limitations of conducting an IPD meta-analysis. Even though the raw trial data will be acquired, analysis is dependent on the measures assessed in each original trial, and may be limited. Data harmonisation that is required to conduct an IPD meta-analysis may lead to some data being excluded from the analyses as it is unlikely to be possible to harmonise all data from different measures of each PROGRESS-Plus characteristic. A further limitation is that the estimates of inequality are influenced by the distribution of characteristics within each study. For example, studies with a narrow age range might not identify interactions between intervention effects and age. Finally, we are only looking at UK-based trials of behavioural weight management interventions, which may limit the generalisability of our findings to other countries or healthcare systems.

3.2 Ethics and dissemination

Ethical approval was not required for this study as no primary data are to be collected, and the IPD are to be analysed in accordance to the purpose for which they were originally collected for. Ethical

For peer review only - http://bmjopen.bmj.com/site/about/guidelines.xhtml

approval for each eligible trial for this IPD meta-analysis was obtained by the original investigators of each trial.

We anticipate that the completed IPD meta-analysis will be published in a scientific journal; one collaborator from each trial contributing IPD will be invited to be a co-author on the publication. The findings from this IPD meta-analysis study may also be presented at relevant public health and obesity research conferences, and will contribute to the lead investigator's PhD thesis.

Contributors:

JMB conceived and designed the study, developed the analysis plan, and drafted the manuscript. JM contributed to study design and the analysis plan, and reviewed drafts of the manuscript. SJS contributed to the analysis plan and reviewed drafts of the manuscript. JL, MPK and SJG contributed to study design and reviewed drafts of the manuscript. ALA conceived and designed the study, contributed to the analysis plan, and reviewed drafts of the manuscript. All authors approved the final version for publication.

Funding:

JMB, ALA, SJG, and SJS are supported by the Medical Research Council (MRC) (Grant MC_UU_00006/6). The University of Cambridge has received salary support in respect of SJG from the National Health Service in the East of England through the Clinical Academic Reserve. This work is funded by UKRI grant MC_UU_00006/6.

Conflicts of interest:

JM is a trustee for the Association for the Study of Obesity (unpaid role). JL has received research support from Slimming World and consulting fees from Novo Nordisk and is an employee of

For peer review only

AstraZeneca. This work was performed before she became an AstraZeneca employee and AstraZeneca had no role in the work. MPK has undertaken consultancy for Slimming World and led the obesity and weight management guidelines development for NICE from 2005 until 2014. SJG is principal investigator on a publicly funded (NIHR) trial in which the intervention is provided by WW (formerly Weight Watchers) at no cost. ALA is principal investigator on two publicly funded (NIHR, MRC) trials where the intervention is provided by WW (formerly Weight Watchers) at no cost.

References

1. Newton S, Braithwaite D, Akinyemiju TF. Socio-economic status over the life course and obesity: Systematic review and meta-analysis. *PloS one*. 2017;12(5):e0177151-e0177151.

2. El-Sayed AM, Scarborough P, Galea S. Unevenly distributed: a systematic review of the health literature about socioeconomic inequalities in adult obesity in the United Kingdom. *BMC public health*. 2012;12(1):18.

3. Khanolkar AR, Patalay P. Socioeconomic inequalities in co-morbidity of overweight, obesity and mental ill-health from adolescence to mid-adulthood in two national birth cohort studies. *The Lancet Regional Health – Europe*. 2021;6. doi:10.1016/j.lanepe.2021.100106

4. White M, Adams J, Heywood P. How and why do interventions that increase health overall widen inequalities within populations? In: Babones SJ, ed. *Social Inequality and Public Health*. Policy Press; 2009. https://books.google.co.uk/books?id=NHRoDwAAQBAJ

5. Adams J, Mytton O, White M, Monsivais P. Why Are Some Population Interventions for Diet and Obesity More Equitable and Effective Than Others? The Role of Individual Agency. *PLOS Medicine*. 2016;13(4):e1001990.

6. O'Neill J, Tabish H, Welch V, et al. Applying an equity lens to interventions: using PROGRESS ensures consideration of socially stratifying factors to illuminate inequities in health. *J Clin Epidemiol*. 2014;67(1):56-64.

7. Birch JM, Jones RA, Mueller J, et al. A systematic review of inequalities in the uptake of, adherence to, and effectiveness of behavioral weight management interventions in adults. *Obesity Reviews*. 2022;23(6):e13438. doi:10.1111/obr.13438

8. Weitz E, Kleiboer A, van Straten A, Hollon SD, Cuijpers P. Individual patient data meta-analysis of combined treatments versus psychotherapy (with or without pill placebo), pharmacotherapy or pill placebo for adult depression: a protocol. *BMJ Open*. 2017;7(2):e013478.

9. Stewart LA, Tierney JF. To IPD or not to IPD? Advantages and disadvantages of systematic reviews using individual patient data. *Eval Health Prof*. 2002;25(1):76-97.

10. Chow CK, Islam SMS, Farmer A, et al. Text2PreventCVD: protocol for a systematic review and individual participant data meta-analysis of text message-based interventions for the prevention of cardiovascular diseases. *BMJ Open*. 2016;6(10):e012723.

For peer review only - http://bmjopen.bmj.com/site/about/guidelines.xhtml

For peer review only

11. Stewart LA, Clarke M, Rovers M, et al. Preferred Reporting Items for a Systematic Review and Meta-analysis of Individual Participant Data: The PRISMA-IPD Statement. *JAMA*. 2015;313(16):1657-1665.

12. LeBlanc ES, Patnode CD, Webber EM, Redmond N, Rushkin M, O'Connor EA. Behavioral and Pharmacotherapy Weight Loss Interventions to Prevent Obesity-Related Morbidity and Mortality in Adults: Updated Evidence Report and Systematic Review for the US Preventive Services Task Force. *Jama*. 2018;320(11):1172-1191.

13. Ahern AL, Wheeler GM, Aveyard P, et al. Extended and standard duration weight-loss programme referrals for adults in primary care (WRAP): a randomised controlled trial. *Lancet (London, England)*. 2017;389(10085):2214-2225. doi:https://dx.doi.org/10.1016/S0140-6736(17)30647-5

14. Anderson AS, Craigie AM, Caswell S, et al. The impact of a bodyweight and physical activity intervention (BeWEL) initiated through a national colorectal cancer screening programme: randomised controlled trial. *BMJ*. 2014;348:g1823. doi:10.1136/bmj.g1823

15. Aveyard P, Lewis A, Tearne S, et al. Screening and brief intervention for obesity in primary care: a parallel, two-arm, randomised trial. *The Lancet*. 2016;388(10059):2492-2500. doi:10.1016/S0140-6736(16)31893-1

16. Beeken RJ, Leurent B, Vickerstaff V, et al. A brief intervention for weight control based on habit-formation theory delivered through primary care: results from a randomised controlled trial. *International journal of obesity (2005)*. 2017;41(2):246-254. doi:10.1038/ijo.2016.206

17. Bhopal RS, Douglas A, Wallia S, et al. Effect of a lifestyle intervention on weight change in south Asian individuals in the UK at high risk of type 2 diabetes: a family-cluster randomised controlled trial. *Lancet Diabetes Endocrinol*. 2014;2(3):218-227. doi:10.1016/s2213-8587(13)70204-3

18. Greaves C, Gillison F, Stathi A, et al. Waste the waist: a pilot randomised controlled trial of a primary care based intervention to support lifestyle change in people with high cardiovascular risk. *International Journal of Behavioral Nutrition and Physical Activity*. 2015;12(1):1.

19. Hunt K, Wyke S, Gray CM, et al. A gender-sensitised weight loss and healthy living programme for overweight and obese men delivered by Scottish Premier League football clubs (FFIT): a pragmatic randomised controlled trial. *The Lancet*. 2014;383(9924):1211-1221. doi:10.1016/S0140-6736(13)62420-4

20. Jebb SA, Ahern AL, Olson AD, et al. Primary care referral to a commercial provider for weight loss treatment versus standard care: a randomised controlled trial. *Lancet*. 2011;378(9801):1485-1492. doi:10.1016/s0140-6736(11)61344-5

21. Jolly K, Lewis A, Beach J, et al. Comparison of range of commercial or primary care led weight reduction programmes with minimal intervention control for weight loss in obesity: lighten Up randomised controlled trial. *BMJ*. 2011;343:d6500. doi:10.1136/bmj.d6500

22. Little P, Stuart B, Hobbs FR, et al. An internet-based intervention with brief nurse support to manage obesity in primary care (POWeR+): a pragmatic, parallel-group, randomised controlled trial. *Lancet Diabetes Endocrinol*. 2016;4(10):821-828. doi:10.1016/s2213-8587(16)30099-7

23. Moore H, Summerbell C, Greenwood D, et al. Improving management of obesity in primary care: cluster randomised trial. *BMJ*. 2003;327(7423):1085. doi:10.1136/bmj.327.7423.1085

For peer review only - http://bmjopen.bmj.com/site/about/guidelines.xhtml

24. Nanchahal K, Power T, Holdsworth E, et al. A pragmatic randomised controlled trial in primary care of the Camden Weight Loss (CAMWEL) programme. *BMJ Open*. 2012;2(3):e000793. doi:10.1136/bmjopen-2011-000793

25. Penn L, White M, Oldroyd J, Walker M, Alberti KG, Mathers JC. Prevention of type 2 diabetes in adults with impaired glucose tolerance: the European Diabetes Prevention RCT in Newcastle upon Tyne, UK. *BMC Public Health*. 2009;9:342. doi:10.1186/1471-2458-9-342

26. Sniehotta FF, Evans EH, Sainsbury K, et al. Behavioural intervention for weight loss maintenance versus standard weight advice in adults with obesity: A randomised controlled trial in the UK (NULevel Trial). *PLoS Med*. 2019;16(5):e1002793. doi:10.1371/journal.pmed.1002793

27. Astbury NM, Aveyard P, Nickless A, et al. Doctor Referral of Overweight People to Low Energy total diet replacement Treatment (DROPLET): pragmatic randomised controlled trial. *BMJ (Clinical research ed)*. 2018;362:k3760. doi:https://dx.doi.org/10.1136/bmj.k3760

28. Daley A, Jolly K, Madigan C, et al. A brief behavioural intervention to promote regular self-weighing to prevent weight regain after weight loss: a RCT. Published online 2019. doi:https://dx.doi.org/10.3310/phr07070

29. Simpson SA, McNamara R, Shaw C, et al. A feasibility randomised controlled trial of a motivational interviewing-based intervention for weight loss maintenance in adults. *Health Technol Assess*. 2015;19(50):v-vi, xix-xxv, 1-378. doi:10.3310/hta19500

30. Sterne JAC, Savović J, Page MJ, et al. RoB 2: a revised tool for assessing risk of bias in randomised trials. *BMJ*. 2019;366:l4898.

31. Jonkman NH, Westland H, Trappenburg JCA, et al. Towards tailoring of self-management for patients with chronic heart failure or chronic obstructive pulmonary disease: a protocol for an individual patient data meta-analysis. *BMJ Open*. 2014;4(5):e005220.

32. Oude Hengel KM, Coenen P, Robroek SJW, et al. Socioeconomic inequalities in reach, compliance and effectiveness of lifestyle interventions among workers: protocol for an individual participant data meta-analysis and equity-specific reanalysis. *BMJ Open*. 2019;9(2):e025463. doi:10.1136/bmjopen-2018-025463

33. Hunter KE, Johnson BJ, Askie L, et al. Transforming Obesity Prevention for CHILDren (TOPCHILD) Collaboration: protocol for a systematic review with individual participant data meta-analysis of behavioural interventions for the prevention of early childhood obesity. *BMJ Open*. 2022;12(1):e048166. doi:10.1136/bmjopen-2020-048166

34. Riley RD, Tierney JF, Stewart LA. *Individual Participant Data Meta-Analysis. A Handbook for Healthcare Research*. 1st ed. Wiley; 2021.

35. NHS Digital. Health Survey for England 2019: Overweight and obesity in adults and children. Published 2020. Accessed October 28, 2022. https://files.digital.nhs.uk/9D/4195D5/HSE19-Overweight-obesity-rep.pdf

36. Booth HP, Prevost AT, Gulliford MC. Access to weight reduction interventions for overweight and obese patients in UK primary care: population-based cohort study. *BMJ Open*. 2015;5(1):e006642. doi:10.1136/bmjopen-2014-006642

For peer review only

37. Coulman KD, Redaniel T, Margelyte R, et al. *Patterns of Adult Weight Management Referrals in Primary Care in England*. University of Bristol; 2022.

38. Birch JM, Griffin SJ, Kelly MP, Ahern AL. Inequalities in the uptake of, adherence to and effectiveness of behavioural weight management interventions: systematic review protocol. *BMJ Open*. Published online 2020. doi:10.1136/bmjopen-2020-039518

For peer review only

For peer review only - http://bmjopen.bmj.com/site/about/guidelines.xhtml

Table 1 Summary of eligible studies for inclusion in IPD meta-analysis

Study name (first author)	Year published	Intervention group description	Control group description	Participant eligibility criteria
**WRAP (Ahern)**	2017	Participants were given vouchers to attend WW (previously Weight Watchers) meetings once a week and access WW digital tools for the duration of their intervention (12- or 52-weeks) for free.	A printed booklet of self-help weight-management strategies (British Heart Foundation).	Inclusion: Aged $\geq$18 years and BMI $\geq$28 kg/m^2.<br><br>Exclusion: Planned or current pregnancy, previous or planned bariatric surgery, current participation in a weight-loss programme, having an eating disorder, non-English speaking.
**BeWEL (Anderson)**	2014	12-month intervention delivered by trained lifestyle counsellors in 3 x 1-hour one-to-one visits during the first 3 months, followed by 9 monthly 15-minute telephone conversations, leading to a total contact time of 5.25 hours.	A printed booklet of self-help weight-management strategies (British Heart Foundation).	Inclusion: Aged 50 to 74 years, had undergone polypectomy for adenoma, and BMI >25 kg/m^2.<br><br>Exclusion: Pregnancy, insulin dependent diabetes mellitus, and any cancer diagnosis.
**BWeL (Aveyard)**	2016	General Practitioners offered participants referral to a commercial weight management programme of 12 1-hour sessions (Slimming World, Rosemary Conley) and gave vouchers to allow them to attend for free.	General Practitioners advised participants to lose weight	Inclusion: Aged $\geq$ 18 years, BMI $\geq$25 kg/m^2 (if Asian ethnicity) or BMI $\geq$30 kg/m^2 (if other ethnicities), have a raised body fat percentage.<br><br>Exclusion: Planned or current pregnancy, previous bariatric surgery, completed or participating in a weight management programme within previous 3 months, non-English speaking.
**Ten Top Tips *[10TT]* (Beeken)**	2017	10TT was a self-guided leaflet-based intervention that used habit-formation theory to aid weight loss. A logbook was provided for participants to self-monitor target behaviours.	Usual care, dependent on the participant's General Practitioner. May include dietary advice or referral to a commercial programme.	Inclusion: Aged $\geq$ 18 years, BMI $\geq$ 30 kg/m^2.<br><br>Exclusion: Unable to provide informed consent due to mental incapacity or active psychotic illness, pregnant, or terminally ill.

**PODOSA (Bhopal)**	2014	15 visits from a dietitian over 3 years, where the dietitian would advise participants on achieving weight loss through culturally adapted and translated resources.	4 visits from a dietitian over 3 years where standard advice on healthy eating, diabetes prevention and physical activity was given.	Inclusion: Aged ≥ 35 years, self-identified men and women of Indian or Pakistani origin with waists measuring ≥ 90cm (men) or ≥ 80cm (women), impaired glucose tolerance or impaired fasting glucose tolerance, the family cook was cooperative.<br><br>Exclusion: Receiving long-term oral corticosteroids or weight loss medication, having long-term health disorders making adherence improbable, pregnant, and unlikely to remain in the UK for 3 years.
**Waste the Waist (Greaves)**	2015	4 x 2-hour group-based sessions in the first month to support behaviour change for weight loss, then 5 x 90-minute group sessions over the next 8 months to support maintenance of behaviour change, totalling 13.5 hours of contact time.	Participants were provided written information on the effects of diet and physical activity on cardiovascular risk.	Inclusion: Aged 40-74 years, BMI ≥ 28 kg/m^2, and having a high cardiovascular risk defined using either the Framingham or QRISK2 algorithm.<br><br>Exclusion: Existing heart disease, type 2 diabetes mellitus, BMI > 40 kg/m^2,
**Football Fans in Training (Hunt)**	2014	12 weekly sessions of 90-minutes in length, delivered at 13 Scottish professional football club stadiums. Each 90 min session combined advice on healthy diet with physical activity. The balance of classroom and physical activity sessions changed during the 12 weeks; later weeks focused on physical activity as men became fitter, and the shorter classroom sessions focused on revision. The 12-week active phase was followed by a weight maintenance phase with six post-	12 month waiting list to receive the FFIT intervention.	Inclusion: Men, aged 35-65 years, BMI ≥ 28 kg/m^2, completed physical activity readiness questionnaire, not taken part in FFIT previously.<br><br>Exclusion: Blood pressure that contraindicated vigorous exercise (systolic ≥160 mm Hg or diastolic ≥100 mm Hg) were excluded from the more intense physical activity programme sessions.

		programme email prompts during 9 months and a group reunion at the club 6 months after the end of the sessions.		
**(Jebb)**	2011	Participants were given vouchers to attend WW (previously Weight Watchers) meetings once a week for 12 months and access digital tools for free.	Advice from their GP and other standard care in line with national treatment guidelines.	Inclusion: Aged > 18 years, BMI 27-35 kg/m^2, and at least one risk factor for obesity related disease (such central adiposity, type 2 diabetes mellitus without inulin treatment, family history of diabetes).<br><br>Exclusion: Achieved weight loss of > 5kg in previous 3 months, history of clinically diagnosed eating disorder, orthopaedic limitations preventing regular physical activity, untreated thyroid disease or more than one change in thyroid treatment in the previous 6 months; receiving treatment with effects on weight or appetite; gastrointestinal disorders; previous surgical procedure for weight loss; major surgery in the previous 3 months; pregnancy or lactation; insulin-treated diabetes; diabetes diagnosis in the previous 6 months; glycated haemoglobin (HbA1c) of at least 75 mmol/mol (9·0%); heart problems in the previous 3 months; uncontrolled hypertension; new prescription drug for a chronic disorder in the previous 3 months or change in dose in the previous 1 month; history or presence of cancer, with the exception of completely resected basal or squamous cell carcinoma if treatment completed 6 months before enrolment or if treatment was stable; or participation in another clinical trial in the previous 30 days.
**Lighten Up (Jolly)**	2011	In addition to 12 vouchers for free entrance to a local leisure centre, participants were randomised to one of 7 intervention groups (all 12 weeks in	12 vouchers for free entrance to a local leisure centre.	Inclusion: Registered with general practices in the South Birmingham Primary Care Trust, aged > 18 years, had a raised BMI recorded in primary care notes within previous 15 months (White Europeans and all ethnic groups apart from South Asians

For peer review only

		length): Weight Watchers, Slimming World, Rosemary Conley, Size Down, GP-led one-to-one counselling, pharmacy-led one-to-one counselling, or a choice of any of the six intervention programmes.		with no comorbidities BMI 30 ≥ kg/m^2, White Europeans and all ethnic groups apart from South Asians with comorbidities BMI 28 ≥ kg/m^2, South Asians with no comorbidities 25 ≥ kg/m^2, South Asians with comorbidities 23 ≥ kg/m^2).<br><br>Exclusion: Unable to understand English or were pregnant.
**POWeR+ (Little)**	2016	Two intervention groups, both consisting of a 24-session web-based weight management programme lasting 6 months.<br><br>POWeR+F provided three scheduled (and four optional) face-to-face nurse support sessions.<br><br>POWeR+R included three phone or email contacts and two optional phone or email contacts.	Brief advice web-pages for a healthier diet.	Inclusion: Aged ≥ 18 years and BMI ≥ 30 kg/m2 or more (or ≥ 28 kg/m2 with hypertension, hypercholesterolaemia, or diabetes).<br><br>Exclusion: Severe mental health problems, too ill to participate in the study or unable to change diet due to health, pregnant or breastfeeding, perceived inability to walk 100m, another member of household participating, no regular access to the internet.
**(Moore)**	2003	Intervention was targeted at general practitioners and practice nurses, and the unit of randomisation was primary care practice. The intervention consisted of 3x90-minute sessions that trained the practitioners on a model approach to obesity treatment.	Control practices were asked to provide usual care to patients.	Inclusion: Aged 16-64 years and BMI ≥ 30 kg/m^2.
**CAMWEL (Nanchahal)**	2012	One-to-one programme delivered across 14 visits over 12 months by advisors trained in obesity causes, diet and physical activity, behaviour change strategies,	Usual care.	Inclusion: Aged ≥ 18 years and BMI ≥25 kg/m^2.

For peer review

		motivational interviewing and cognitive behavioural therapy techniques.		
**EDIPS (Penn)**	2009	Participants received regular individual advice from a dietitian and physiotherapist trained in motivational interviewing. Participants were also invited to group events, such as 'cook and eat'. Individual sessions were for 30 minutes monthly for the first 3 months and then every 3 months for up to 5 years.	Brief advice and usual care from GP.	Inclusion: Aged >40 years, BMI > 25kg/m^2, and impaired glucose tolerance of ≥ 7.8 mmol/l and < 11.1 mmol/l.<br><br>Exclusion: Previous diagnosis of diabetes mellitus, chronic illness that makes participation in moderate physical activity impossible, or on a special diet for medical reasons.
**NULevel (Sniehotta)**<br><br>***Weight loss maintenance***	2019	Intervention was delivered via a combination of a single face-to face meeting and regular text messaged (at least 1 every 2 days). The text messages consisted of content that was triggered by participants daily self-weighing and questionnaire completion.	Brief lifestyle advice received by text message on 4 occasions, 3 months apart.	Inclusion: Aged $\geq$ 18 years, BMI $\geq$ 30kg/m^2 ($\geq$ 28kg/m^2 if of South Asian descent) in the 24 months preceding trial, and had lost $\geq$5% of body weight in the preceding 12 months.<br><br>Exclusion: Lost weight through illness or surgical procedures, pregnant or planning to become pregnant during study period, breastfeeding, unable to understand English, diagnosis of an eating disorder or condition that limits physical activity, or plans to leave geographic area during study period.
**DROPLET (Astbury)**	2018	The intervention was a Total Diet Replacement programme, which consisted of weekly behavioural support for 12 weeks and monthly support for 3 months with formula food products providing 810 kcal per day for the first 8 weeks, followed by gradual reintroduction of food.	Behavioural support for weight loss from a practice nurse and a diet programme with modest energy restriction.	Inclusion: Aged $\geq$ 18 years, BMI $\geq$ 30kg/m^2, and participants' GP determines weight loss would benefit health.<br><br>Exclusion: Scheduled or previously received bariatric surgery, currently participating in a weight management programme, and contraindications to total diet replacement.
**LIMIT (Daley)**	2019	Over the course of a 12-week weight maintenance programme, participants carried out daily self-weighting, received 3	Brief advice leaflet.	Inclusion: Aged $\geq$ 18 years, had lost $\geq$ 5% of their weight by the end of their weight loss programme, owned a mobile or landline

For peer review only

Weight loss maintenance		brief support phone calls delivered by non-specialist call centre staff, and text messages sent every other day for the first 4 weeks and twice weekly for the remaining 8 weeks.		phone that could receive text messages, was able to understand English sufficiently to complete study procedures.<br><br>Exclusion: Pregnant or intending to become pregnant during the study period.
**WILMA (Simpson)**<br><br>***Weight loss maintenance***	2015	Two intervention groups, both comprising of a 12-month intervention. Participants in both intervention groups could attend 4 peer group support sessions lasting 1.5 hours for 4 months following the face-to-face sessions.<br><br>Intensive group – participants received 6 one-to-one face-to-face individually tailored motivational interviewing delivered fortnightly for 3 months (each session listing around 60 minutes). In the remaining 9 months of the intervention, participants received monthly motivational interviewing calls lasting around 20 minutes.<br><br>Less intensive group – participants received two face-to-face motivational interviewing sessions two weeks apart and two motivational interviewing phone calls at 6-months and 12-months.	Brief advice leaflet.	Inclusion: Aged 18-70 years, current or previous BMI of ≥ 30 kg/m^2, and intentionally lost ≥5% of body weight in previous 12 months.<br><br>Exclusion: Previous bariatric surgery (unless reversed), terminal illness, inability to understand study materials in English, living with another study participant, or currently pregnant.
